# FGF21-FGFR1 controls mitochondrial homeostasis in cardiomyocytes by modulating the degradation of OPA1

**DOI:** 10.1038/s41419-023-05842-9

**Published:** 2023-05-08

**Authors:** Bing Yan, Zhu Mei, Yaohan Tang, Haixu Song, Hanlin Wu, Quanmin Jing, Xiaolin Zhang, Chenghui Yan, Yaling Han

**Affiliations:** 1 National Key Laboratory of Frigid Zone Cardiovascular Disease, Cardiovascular Research Institute and Department of Cardiology, General Hospital of Northern Theater Command, Shenyang, 110016 China; 2 Department of Cardiology, Second Hospital of Jilin University, No. 218 Ziqiang Street, Changchun, 130041 China

**Keywords:** Heart failure, Ubiquitin ligases, Cardiac hypertrophy

## Abstract

Fibroblast growth factor 21 (FGF21) is a pleiotropic hormone secreted primarily by the liver and is considered a major regulator of energy homeostasis. Recent research has revealed that FGF21 could play an important role in cardiac pathological remodeling effects and prevention of cardiomyopathy; however, the underlying mechanism remains largely unknown. This study aimed to determine the mechanism underlying the cardioprotective effects of FGF21. We engineered FGF21 knock out mice and subsequently elucidated the effects of FGF21 and its downstream mediators using western blotting, qRT-PCR, and mitochondrial morphological and functional analyses. FGF21 knockout mice showed cardiac dysfunction, accompanied by a decline in global longitudinal strain (GLS) and ejection fraction (EF), independent of metabolic disorders. Mitochondrial quality, quantity, and function were abnormal, accompanied by decreased levels of optic atrophy-1 (OPA1) in FGF21 KO mice. In contrast to FGF21 knockout, cardiac-specific overexpression of FGF21 alleviated the cardiac dysfunction caused by FGF21 deficiency. In an in vitro study, FGF21 siRNA deteriorated mitochondrial dynamics and impaired function induced by cobalt chloride (CoCl_2_). Both recombinant FGF21 and adenovirus-mediated FGF21 overexpression could alleviate CoCl_2_-induced mitochondrial impairment by restoring mitochondrial dynamics. FGF21 was essential for maintaining mitochondrial dynamics and function of the cardiomyocytes. As a regulator of cardiomyocyte mitochondrial homeostasis under oxidative stress, FGF21 could be an important new target for therapeutic options for patients with heart failure.

## Introduction

Fibroblast growth factor 21 (FGF21) is a well-known endocrine hormone that increases fat mobilization and insulin sensitivity [1–4]. FGF21 regulates metabolic homeostasis mainly through FGFR1 [5]. Considering the close association between metabolism and the cardiovascular system, studies have investigated the cardiac effects of FGF21. The results showed that FGF21 could protect against hypertrophic cardiomyopathy [6–8], which is the early stage of cardiac dysfunction, in a mouse model [9]. However, there are conflicting results in clinical research, which show that increased serum FGF21 in HFrEF patients is correlated with worse outcomes [10, 11]. However, whether FGF21 has a direct protective effect on cardiac function remains unknown.

The mitochondria are important organelles that maintain cellular energy production and other biological processes [12]. They can adapt to a variety of endogenous and exogenous stimuli mainly through dynamic changes in fission and fusion [13]; therefore, mitochondrial dynamics is a vital mechanism for damage protection [14]. Damaged mitochondria are closely associated with cardiac dysfunction [15–17]. Optic Atrophy 1 (OPA1) is a dynamin-like GTPase located in the inner mitochondrial membrane that regulates mitochondrial fusion and maintains mitochondrial cristae structure [18, 19]. OPA1 also plays an important role in maintaining mitochondrial function by regulating the respiratory supercomplex assembly and modulating mitochondrial calcium uptake [20, 21].

To elucidate the effect of FGF21 on cardiac function, we focused on the effects of FGF21 on cardiac function. In this study, we observed cardiac dysfunction in FGF21 KO mice, and found that FGF21 deficiency can cause severe mitochondrial damage, both in vivo and in vitro. Both exogenous addition and endogenous overexpression alleviated mitochondrial damage caused by Cobalt chloride (CoCl_2_) stimulation. Therefore, we further investigated the mechanism by which FGF21 maintains mitochondrial homeostasis and found that FGF21 inhibits OPA1 degradation by activating FGFR1 and increasing FBXW11 degradation.

## Results

### FGF21 KO mice developed cardiac dysfunction before onset of impaired metabolic state

**Fig. 1:**
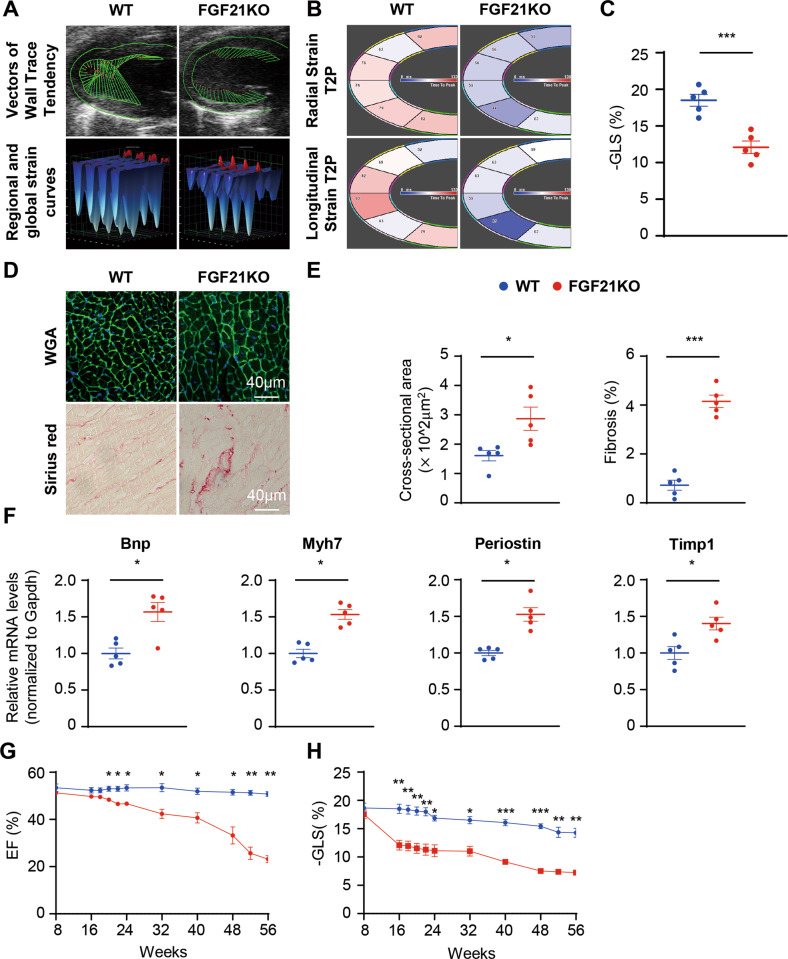
16-week-old FGF21 KO mice developed diastolic dysfunction, mild cardiac hypertrophy, and fibrosis. **A** Representative 16-week WT and FGF21 KO mice echocardiography images of vectors of wall trace tendency calculated by Vevo Software. **B** Representative 16-week WT and FGF21 KO mice echocardiography images of vectors of radial and longitudinal strain calculated by Vevo Software. **C** 16-week WT and FGF21 KO mice left ventricular global longitudinal strain (GLS) was calculated by Vevo Software. *n* = 5 mice. **D** Representative cross-sections of heart stained for WGA, and Sirius red in 16-week WT and FGF21 KO mice heart tissue. **E** Quantitative analysis of cardiomyocyte size (WGA staining), fibrotic area (red area in Sirius red staining). *n* = 5 mice. **F** Relative mRNA levels of hypertrophic and fibrotic genes in the 16-week WT and FGF21 KO mice heart. *n* = 5 mice. **G**, **H** Ejection fraction and GLS were measured by echocardiography every 8 weeks (every 2 weeks in 16–24 week, every 4 weeks in 48–56 week) in WT and FGF21 KO mice. *n* = 5 mice. Data are expressed as the mean ± SEM, with individual data points. Data were analyzed by two-tailed unpaired Student’s test (**C**, **E**, **F**), two-way RM ANOVA with Geisser-Greenhouse’s correction (**G**, **H**).

To understand the mechanism underlying the beneficial effect of FGF21 on cardiovascular health, we used FGF21 KO mice (Fig. S1A–D), and closely monitored the metabolic baseline data and cardiac function. Unexpectedly, although FGF21 is a key regulator of metabolism, there were significant differences in cardiac diastolic function between FGF21 KO mice and their littermates at 16 weeks old, but not in blood glucose, body weight, or insulin sensitivity, as evidenced by comparable global longitudinal strain(GLS), and longitudinal and radial strain time to peak (Fig. 1A–C, and Fig. S1E–J). In 16-week-old FGF21 KO hearts, mild hypertrophic features and increased cardiac fibrosis were observed (Fig. 1D, E). The cardiac impairment marker Bnp, hypertrophy marker Myh7, and fibrosis markers Periostin and Timp1 were also increased (Fig. 1F). Although the ejection fraction was not significantly impaired in 16-week-old FGF21 KO mice, severe systolic dysfunction developed after 22 weeks of age (Fig. 1G, H), with increased blood glucose, body weight, and impaired insulin sensitivity (Fig. S1E–J). These results suggest that FGF21 plays a key role in maintaining the cardiac function and metabolic status.

### FGF21 depletion led to defective mitochondrial morphology and reduced OPA1 levels

**Fig. 2:**
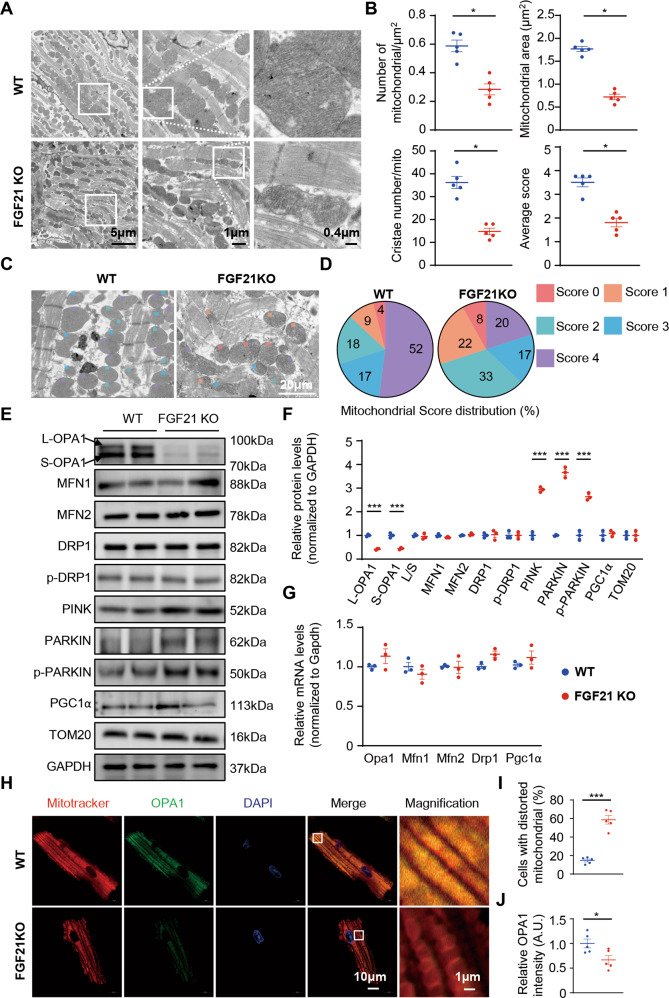
In FGF21 KO mice, mitochondrial morphology is severely damaged. **A** Representative electron microscopic (EM) images of 16-week WT and FGF21 KO mice heart sections. **B** Quantification of number of mitochondria per square micron, average mitochondria cross-sectional area, cristae number, and average mitochondria score based on a 5-grade scoring system. Per heart, 15 to 20 random fields were analyzed. *n* = 5 mice. **C** Representative EM images of 16-week WT and FGF21 KO mice heart sections marked by a 5-grade scoring system. **D** Pie chart depicts the prevalence of the individual score grades across the analyzed mitochondrial population in each group. A total of 100 mitochondria in each sample were randomly analyzed. *n* = 5 mice. **E**, **F** Representatives immunoblot images of total proteins extracted from the 16-week WT and FGF21 KO mice heart tissues and quantified by densitometric analysis. *n* = 5 mice. **G** Relative mRNA levels of the mitochondrial dynamic-related protein and mitochondrial genesis protein in the heart of 16-week WT and FGF21 KO mice. *n* = 5 mice. **H**–**J** Representative confocal images and quantification of the 16-week WT and FGF21 KO mice isolated adult cardiomyocytes MitoTracker and OPA1 staining. Isolated adult cardiomyocytes (>20) were evaluated mitochondrial morphology to determine the mitochondria is normal or distorted of each heart. *n* = 5 mice. Data are expressed as the mean ± SEM, with individual data points. Data were analyzed by two-tailed unpaired Student’s t test (**B**, **D**, **F**, **G**, **I**, **J**).

To investigate how FGF21 deficiency mediates cardiac dysfunction, we examined the morphology and function of heart tissue in FGF21 KO mice and their littermates at 16 weeks old. TEM analysis demonstrated that sarcomere arrangement and mitochondrial morphology were disrupted in FGF21 KO mice (Fig. 2A). The number of mitochondria per square micron and mean mitochondrial area were significantly reduced in FGF21 KO mice (Fig. 2B), which also indicated a reduction in mitochondrial mass. FGF21 KO heart mitochondria contained fewer cristae than WT heart mitochondria (Fig. 2B). Analysis with the 5-grade scoring system showed that FGF21 deficiency induced severe damage in mitochondrial morphology with inflation, warped membranes, irregularities, and absence of cristae (Fig. 2B–D and Fig. S2A).

To elucidate the mechanism of mitochondrial damage, we further investigated mitochondrial dynamic-related proteins, mitochondrial genesis-associated proteins, and indicators of mitophagy. No difference was observed in mitochondrial fusion-related proteins MFN1 and MFN2, and fission-related protein DRP1 and activated DRP1 (Ser 616 phosphorylation) in 16-week-old WT and FGF21 KO mice. The expression of the mitochondrial genesis-associated protein PGC1α was also the same in both groups. However, mitochondrial fusion-related protein OPA1 was significantly decreased, and mitophagy-related proteins PINK, PARKIN, and p-PARKIN (Ser65) were significantly increased in FGF21 KO mice (Fig. 2E, F).

Eight OPA1 variants are present in humans, and five variants are present in mice. In mice, OPA1 can be resolved into five bands ranging in size from 85 to 100 kD. The bands are labeled ‘a–e’ and correspond to different isoforms. The a and b bands are classified into long isoforms (L-OPA1), and the c, d, and e bands are classified into short isoforms (S-OPA1). L-OPA1 promotes mitochondrial fusion and S-OPA1 promotes fission. Both total OPA1 (T-OPA1) and the balance between L-OPA1 and S-OPA1 can affect mitochondrial dynamics [21, 22]. In the hearts of FGF21 KO mice, total OPA1 decreased, with L-OPA1 and S-OPA1 decreasing in equal proportions, indicating that FGF21 had no effect on the ratio between L-OPA1 and S-OPA1 (Fig. 2F). To further clarify whether FGF21 deficiency affects the balance between L-OPA1 and S-OPA1, we evaluated the levels of OMA1 and YME1L, mitochondrial proteases that cleave OPA1 from L-OPA1 to S-OPA1. The results showed that loss of FGF21 had no effect on OMA1 and YME1L levels (Fig. S3A).

To confirm whether mitophagy was active, we evaluated p-PARKIN recruitment in the mitochondria by immunofluorescent staining of isolated adult cardiomyocytes of 16-week-old WT and FGF21 KO mice (Fig. S3B, C). The results showed increased recruitment of p-PARKIN in FGF21 KO mice, indicating activation of mitophagy. We further investigated the mRNA levels of related proteins and found no differences between the FGF21 KO mice and their littermates (Fig. 2G).

To further investigate whether the decreased OPA1 impacted mitochondrial dynamics in cardiomyocytes, we used MitoTracker staining to evaluate mitochondrial morphology in isolated adult cardiomyocytes of 16-week-old WT and FGF21 KO mice. As expected, FGF21 KO adult cardiomyocyte mitochondria were characterized by increased splitting, swelling, and disorganization (Fig. 2H–J). These results suggested that FGF21 deficiency may cause reduced OPA1 levels and mitochondrial damage.

### Restoration of cardiac FGF21 reversed impaired cardiac function in FGF21 KO mice

**Fig. 3:**
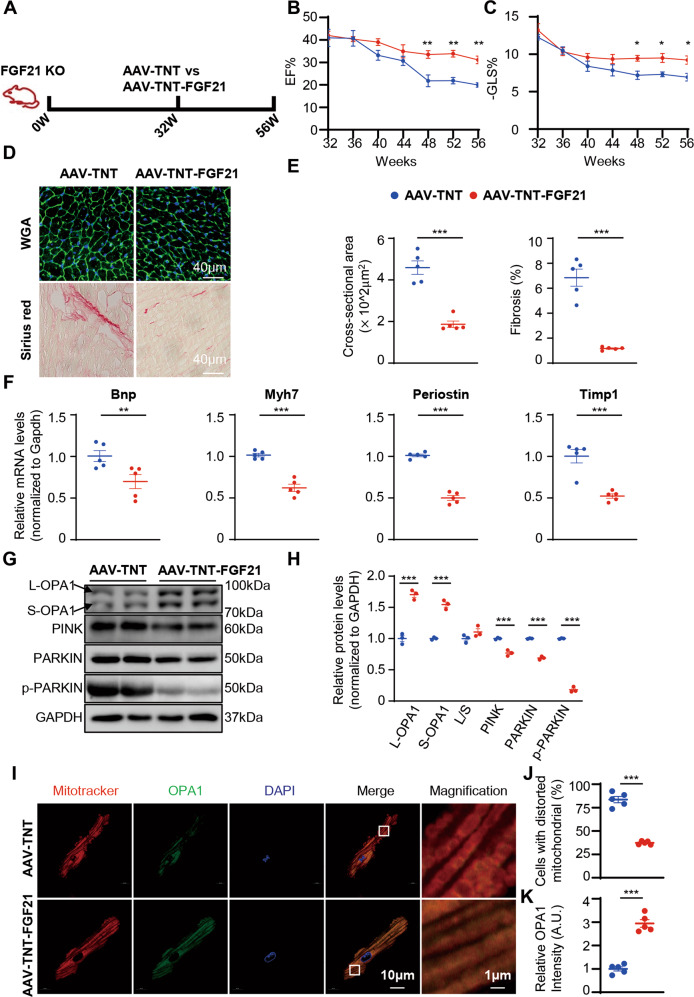
Cardiac-specific overexpression of FGF21 could reduce cardiac dysfunction in FGF21 KO mice. **A** The strategy for conducting cardiac-specific overexpression FGF21 mice. 32-week-old FGF21 KO mice and their wild-type littermates were given AAV-TNT-FGF21 and AAV-TNT, then noninvasive echocardiographic assessments were performed to monitor cardiac function for 24 weeks. **B** Ejection fraction was measured by echocardiography every 4 weeks in AAV-TNT and AAV-TNT-FGF21 mice. *n* = 5 mice. **C** Left ventricular global longitudinal strain was calculated by Vevo Software every 4 weeks. *n* = 5 mice. **D** Representative cross-sections of heart stained for WGA, and Sirius red in AAV-TNT and AAV-TNT-FGF21 mice heart tissue. **E** Quantitative analysis of cardiomyocyte size (WGA staining), fibrotic area (red area in Sirius red staining). *n* = 5 mice. **F** Relative mRNA levels of hypertrophic and fibrotic genes in the heart. *n* = 5 mice. **G**, **H** Representative immunoblot images of total proteins extracted from the heart tissues and quantified by densitometric analysis. *n* = 5 mice. **I**–**K** Representative confocal images and quantification of isolated adult cardiomyocytes MitoTracker and OPA1 staining. Isolated adult cardiomyocytes (>20) were evaluated mitochondrial morphology to determine the mitochondria is normal or distorted of each heart. *n* = 5 mice. Data are expressed as the mean ± SEM, with individual data points. Data were analyzed by two-way RM ANOVA with Geisser-Greenhouse’s correction (**B**, **C**), two-tailed unpaired Student’s t test (**E**, **F**, **H**, **J**, **K**).

To confirm that FGF21 exerts cardiac-specific protection against mitochondrial damage, an adeno-associated virus carrying a TNT promoter (AAV-TNT) was used to conduct FGF21 cardiac-specific overexpression in FGF21 KO mice (Fig. 3A and Fig. S4A). FGF21 cardiac-specific overexpression had no effect on metabolic status (Fig. S4B, C). But as expected, FGF21 KO mice injected with AAV-TNT-FGF21 showed reduced the cardiac dysfunction comparing with FGF21 KO mice injected with AAV-TNT, as shown by the ejection fraction (EF) and global longitudinal strain (GLS) (Fig. 3B, C). Consistently, FGF21 cardiac-specific overexpression reversed the cardiac hypertrophy and fibrosis caused by FGF21 deficiency (Fig. 3D, E). The cardiac impairment marker Bnp, hypertrophy marker Myh7, and fibrosis markers Periostin and Timp1 were also decreased in FGF21 cardiac-specific overexpression mice (Fig. 3F). Western blotting showed that OPA1 levels was increased. The reduction in PINK, PARKIN, and p-PARKIN (Ser65 phosphorylation) indicated that mitophagy was inhibited in FGF21 cardiac-specific overexpression mice (Fig. 3G, H). To confirm whether mitophagy was inhibited, we evaluated p-PARKIN recruitment in mitochondria by immunofluorescence analysis of isolated adult cardiomyocytes (Fig. S4D, E). The results showed decreased recruitment of p-PARKIN in FGF21 cardiac-specific overexpression mice, indicating the inhibition of mitophagy.

Furthermore, MitoTracker staining of FGF21 KO mouse mitochondria in isolated adult cardiomyocytes revealed that FGF21 cardiac-specific overexpression reversed the decreased OPA1 levels, also the increased mitochondrial splitting and swelling (Fig. 3I–K). These results demonstrated that FGF21 cardiac-specific overexpression could reverse cardiac dysfunction caused by FGF21 deficiency.

### FGF21 deficiency caused decreased OPA1 levels and abnormal mitochondrial structure and function in vitro

**Fig. 4:**
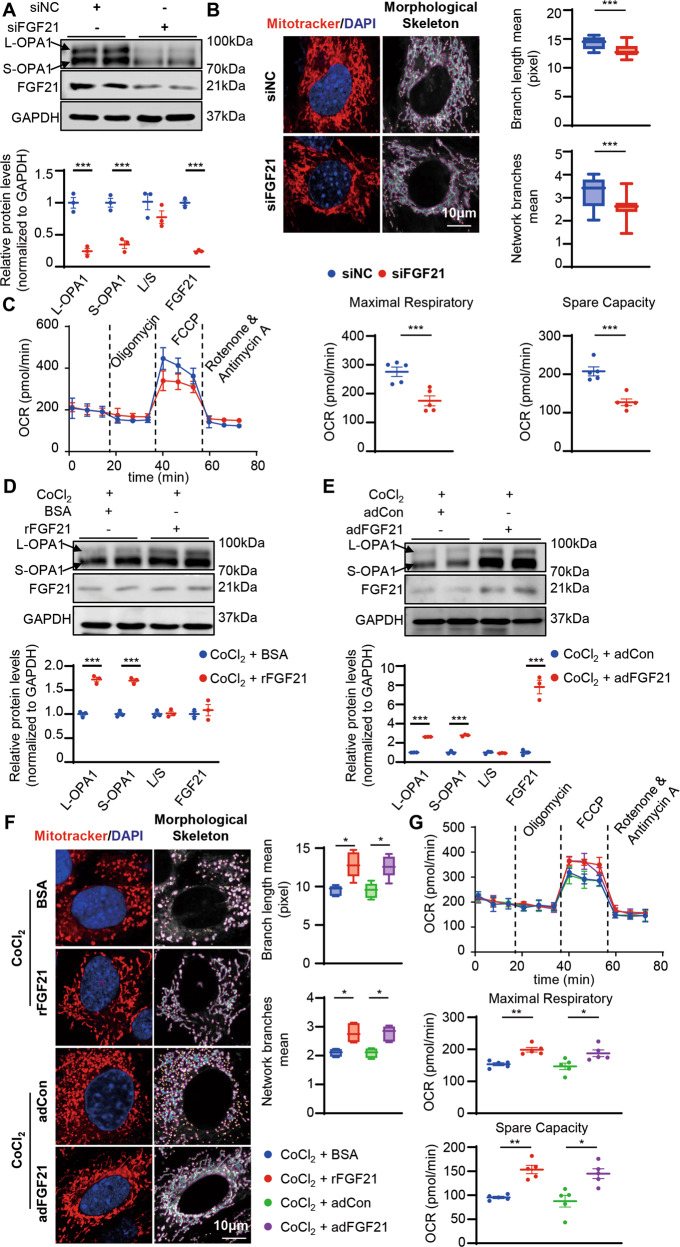
FGF21 knocked down cardiomyocytes had abnormal mitochondrial structure and function, and overexpression FGF21 could protect against mitochondrial damage. **A** Representatives immunoblot images and quantification of the OPA1 and FGF21 in neonatal mouse cardiomyocytes treated with non-targeting siRNA (siNC) or siRNA targeting Fgf21 (siFGF21). *n* = 3. **B** Representative images of mitochondrial network visualized by Mitotracker staining (Mitotracker in red and DAPI in blue) in neonatal mouse cardiomyocytes treated with siNC or siFGF21 and mitochondrial morphological skeleton generated by MiNA (a ImageJ macro tool), and quantification of mitochondrial network. Branch length and network branches was analyzed by MiNA in ImageJ. Summary statistics for all cells (30 cells from 3 experiments were analyzed, 10 cells per experiment), box plots show median (horizontal lines), first to third quartile (box), and the most extreme values (vertical lines). **C** Representative oxygen consumption curves in the siFGF21 and siNC treated HL-1 cells. Basal respiration rate was measured followed by proton leak after the addition of oligomycin (1.5 μM), maximal respiration was measured after the addition of FCCP (0.5 μM), and non-mitochondrial respiration after the addition of rotenone and antimycin (0.5 μM). Quantification analysis of maximal respiratory (defined as the difference between FCCP-stimulated OCR and non-mitochondrial respiration) and spare capacity (defined as the difference between maximal respiration and basal respiration). *n* = 5 per group. **D**, **E** Representatives immunoblot images and quantification of the OPA1 and FGF21 in neonatal mouse cardiomyocytes treated with rFGF21 (50 ng/ml) or adFGF21 (transfected 24 h) then treated with CoCl_2_ (200 nm, 24 h). *n* = 3. **F** Representative images of mitochondrial network visualized by Mitotracker staining (Mitotracker in red and DAPI in blue) in neonatal mouse cardiomyocytes treated with rFGF21 (50 ng/ml) or adFGF21 (transfected 24 h) then treated with CoCl_2_ (200 nm, 24 h) and mitochondrial morphological skeleton generated by MiNA (a ImageJ macro tool). Quantification of mitochondrial network, branch length and network branches was analyzed by MiNA in ImageJ. Summary statistics for all cells (30 cells from 3 experiments were analyzed, 10 cells per experiment), box plots show median (horizontal lines), first to third quartile (box), and the most extreme values (vertical lines). **G** Representative oxygen consumption curves in HL-1 cells treated with rFGF21 (50 ng/ml) or adFGF21 (transfected 24 h) then treated with CoCl_2_ (200 nm, 24 h). Basal respiration rate was measured followed by proton leak after the addition of oligomycin (1.5 μM), maximal respiration was measured after the addition of FCCP (0.5 μM), and non-mitochondrial respiration after the addition of rotenone and antimycin (0.5 μM). Quantification analysis of maximal respiratory and spare capacity. *n* = 5 per group. Data are expressed as the mean ± SEM, with individual data points (**A**, **D**, **E**), and the mean ± SD (**C**, **G**). Data were analyzed by two-tailed unpaired Student’s t test (**A**–**E**), ordinary one-way ANOVA with Tukey’s multiple comparisons test (**F**, **G**).

To further explore the role of FGF21 in maintaining mitochondrial homeostasis, cardiomyocytes were transfected with siFGF21. As expected, FGF21 deficiency resulted in decreased OPA1 levels (Fig. 4A). Mitotracker staining showed increased fission with decreased mitochondrial network branches and branch length in cardiomyocytes transfected with siFGF21 (Fig. 4B). To validate whether FGF21 deficiency affected mitochondrial oxidative phosphorylation, we used an extracellular flux analyzer to assess mitochondrial function in cardiomyocytes. FGF21-deficient cardiomyocytes had similar levels of basal respiration and proton leak as normal cardiomyocytes, but the maximal respiratory capacity and spare capacity were markedly reduced (Fig. 4C).

### Overexpression of FGF21 had no effect on physiological mitochondrial function

Since the role of FGF21 in maintaining mitochondrial homeostasis was confirmed, we explored whether FGF21 could regulate mitochondrial function under basal conditions. We further evaluated the mitochondrial dynamics and function after administration of recombinant FGF21 (rFGF21) or adenovirus vector encoding FGF21 (adFGF21). Under basal conditions, the rFGF21 or adFGF21 had no effect on mitochondrial dynamics and function (Fig. S5 A–J).

### Endogenous and exogenous FGF21 provided defense against mitochondrial damage under stress in vitro

To further explore whether FGF21 could defend against mitochondrial damage under stressful conditions, we applied different stimulants and observed subsequent FGF21 and OPA1 expression in cardiomyocytes. Cobalt chloride (CoCl_2_), a well-known chemical inducer of HIF1α, has been extensively used as a hypoxia-mimicking agent in both in vitro and in vivo studies [23]. We also used H_2_O_2_ to mimic oxidative stress, phenylephrine (PE), and angiotensin II (AngII) to prepare a cardiac hypertrophic model in vitro [24], and palmitic acid (PA) to induce lipotoxicity [25]. CoCl_2_ activated HIF1α expression and decreased FGF21 and OPA1 levels in a dose- and time-dependent manner, indicating that the hypoxic effect caused by CoCl_2_ was closely related to FGF21 and OPA1 levels (Fig. S6A–F). CoCl_2_ caused L-OPA1 and S-OPA1 to decrease in equal proportions, indicating that CoCl_2_ decreased total OPA1 with no effect on the ratio of L-OPA1 to S-OPA1 (Fig. S6C–F). Moreover, CoCl_2_ stimulation also increased mitochondrial fission and impaired mitochondrial function (Fig. S7A–E). Based on these results, we chose CoCl_2_ as a pathological stimulator to investigate the protective mechanism of FGF21.

Under CoCl_2_ stimulation, recombinant FGF21 and adFGF21 both restored OPA1 levels (Fig. 4D, E). We further investigated mitochondrial morphology; recombinant FGF21 or adFGF21 both restored mitochondrial dynamics and decreased fission, as well as increased fusion and branch formation, under CoCl_2_ stimulation (Fig. 4F). As expected, FGF21 overexpression dramatically improved both spare and maximal respiratory capacity, but did not affect basal respiration, H^+^ leakage, or ATP synthase activity (Fig. 4G).

FGF21 is an endocrine protein that is mainly secreted by the liver; however, cardiomyocytes can also synthesize and secrete FGF21. Significantly high FGF21 levels were observed in adFGF21 cardiomyocyte culture supernatant compared to adenovirus vector (adCon) (Fig. S8A). This result indicated that cardiomyocytes could synthesize and secrete FGF21. Although FGF21 is well-known as a secreted protein, we investigated whether FGF21 could exert biological effects inside the cells. We used monensin, an intracellular protein transport blocker, to block the secretion of FGF21 [26]. When monensin was administered, the high FGF21 levels in the adFGF21 cardiomyocyte culture supernatant decreased. Monensin administration blocked the secretion of FGF21 and compromised the rescue effect of adFGF21 on mitochondrial damage induced by CoCl_2_ (Fig. S8B–G). These results suggest that FGF21 exerts its protective effects mainly through secretion.

### Inhibition of FGFR1 impairs the mitochondrial-protected effect of FGF21 under CoCl_2_ stimulation in vitro

**Fig. 5:**
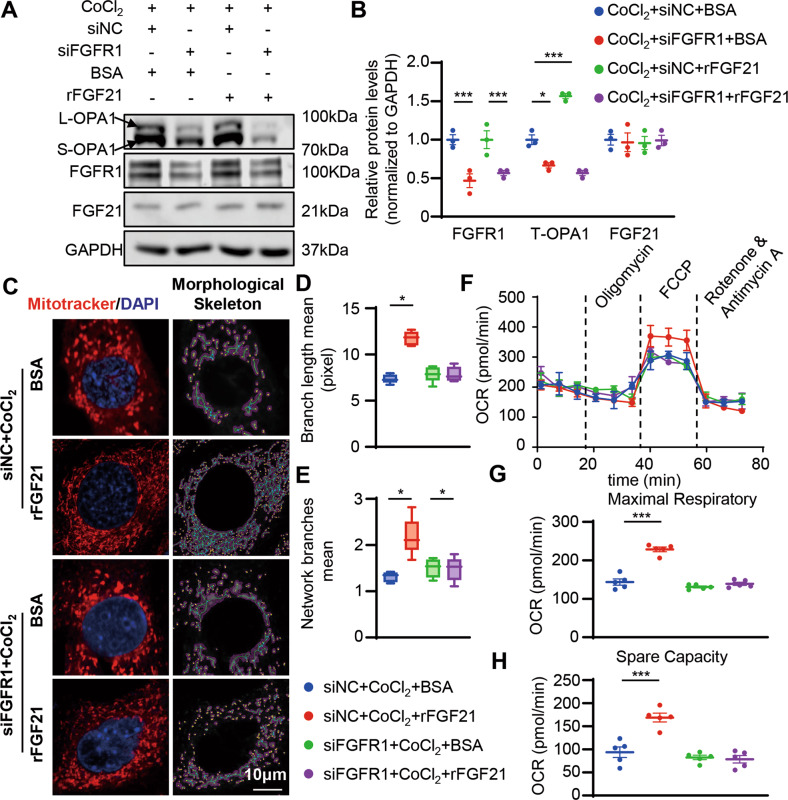
Knocked down FGFR1 impaired the mitochondrial protecting effect of rFGF21 under CoCl_2_ stimulation in vitro. **A**, **B** Representatives immunoblot images and quantification of the OPA1 in siNC or siRNA targeting Fgfr1 (siFGFR1) with or without rFGF21 (50 nM) treated neonatal mouse cardiomyocytes under CoCl_2_ (200 nm, 24 h). *n* = 3 per group. **C** Representative images of mitochondrial network visualized by Mitotracker staining (MitoTracker in red and DAPI in blue) in siNC or siFGFR1 with or without rFGF21 (50 nM) treated HL-1 cells under CoCl_2_ (200 nm, 24 h), and mitochondrial morphological skeleton generated by MiNA (a ImageJ macro tool). **D**, **E** Quantification of mitochondrial network, branch length, and network branches was analyzed by MiNA in ImageJ. Summary statistics for all cells (30 cells from 3 experiments were analyzed, 10 cells per experiment), box plots show median (horizontal lines), first to third quartile (box), and the most extreme values (vertical lines). **F** Representative oxygen consumption curves in siNC or siFGFR1 with or without rFGF21 (50 nM) treated HL-1 cells under CoCl_2_ (200 nm, 24 h). Basal respiration rate was measured followed by proton leak after the addition of oligomycin (1.5 μM), maximal respiration was measured after the addition of FCCP (0.5 μM), and non-mitochondrial respiration after the addition of rotenone and antimycin (0.5 μM). **G**, **H** Quantification analysis of maximal respiratory and spare capacity. *n* = 5 per group. Data are expressed as the mean ± SD (**F**) and the mean ± SEM, with individual data points (**B**, **G**, **H**). Data were analyzed by ordinary one-way ANOVA with Tukey’s multiple comparisons test (**B**, **D**, **E**, **G**, **H**).

As in a previous study [27, 28], FGFR1 and FGFR3 are the main FGF21 receptors in the heart (Fig. S9A). To determine whether FGF21 exerts its effects on the heart through FGFR1 or FGFR3, we transfected cardiomyocytes with either siFGFR1 or siFGFR3, respectively. Only FGFR1 deficiency inhibited OPA1 restoration caused by the recombinant FGF21 and endogenous FGF21 under CoCl_2_ stimulation (Fig. S9B, C, Fig. S10A, B, and Fig. 5A, B). Next, we sought to validate whether FGF21 exerts its effect on the mitochondria by activating FGFR1. As expected, neither endogenous nor exogenous FGF21 could prevent OPA1 degradation in cardiomyocytes when FGFR1 was absent (Fig. 5A, B and Fig. S10A, B). Mitotracker staining showed FGF21 could not restore the decreased mitochondrial fusion caused by CoCl_2_ in FGFR1 absent cardiomyocytes (Fig. 5C–E and Fig. S10C–E). We also tested mitochondrial oxidative phosphorylation in these FGFR1 absent cardiomyocytes. Upregulation of spare and maximal respiratory capacity by FGF21 in CoCl_2_-stimulated cardiomyocytes was absent (Fig. 5F–H and Fig. S10F–H). These results suggested that FGFR1 activation is required for FGF21-mediated mitochondrial protection.

### OPA1-mediated mitochondrial damage caused by FGF21 deficiency or CoCl_2_ stimulation

**Fig. 6:**
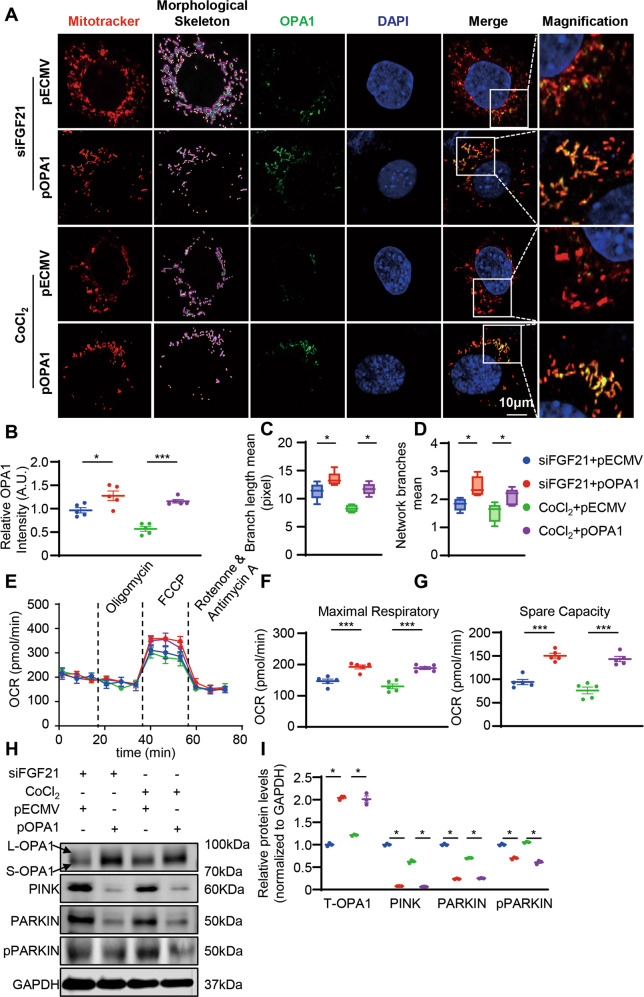
Overexpression of OPA1 restore the excess mitochondrial fission caused by FGF21 deficiency or under CoCl_2_ stimulation. **A**, **B** Representative confocal images and quantification of MitoTracker and OPA1 staining of siFGF21 HL-1 cells then transfect OPA1 plasmid (pOPA1) and HL-1 cells transfected pOPA1 then treated with CoCl_2_ (200 nm 24 h). **C**, **D** Quantification of mitochondrial network, branch length, and network branches was analyzed by MiNA in ImageJ. Summary statistics for all cells (30 cells from 5 experiments were analyzed, 10 cells per experiment), box plots show median (horizontal lines), first to third quartile (box), and the most extreme values (vertical lines). **E** Representative oxygen consumption curves in siFGF21 HL-1 cells then transfect OPA1 plasmid (pOPA1) and HL-1 cells transfected pOPA1 then treated with CoCl_2_ (200 nm 24 h). Basal respiration rate was measured followed by proton leak after the addition of oligomycin (1.5 μM), maximal respiration was measured after the addition of FCCP (0.5 μM), and non-mitochondrial respiration after the addition of rotenone and antimycin (0.5 μM). **F**, **G** Quantification analysis of maximal respiratory and spare capacity. *n* = 5 per group. **H**, **I** Representatives immunoblot images and quantification of the OPA1, PINK, PARKIN, and p-PARKIN in siFGF21 HL-1 cells then transfect OPA1 plasmid (pOPA1) and HL-1 cells transfected pOPA1 then treated with CoCl_2_ (200 nm 24 h). *n* = 3 per group. Data are expressed as the mean ± SD (**E**) and the mean ± SEM, with individual data points (**B**, **F**, **G**, **I**). Data were analyzed by ordinary one-way ANOVA with Tukey’s multiple comparisons test (**B**–**D**, **F**, **G**, **I**).

We aimed to determine whether OPA1 regulation plays an important role in FGF21-induced mitochondrial homeostasis. We overexpressed OPA1 by transfection with an OPA1 plasmid in FGF21 knock down cardiomyocytes or cardiomyocytes under CoCl_2_ stimulation and investigated the mitochondrial morphological changes. Overexpression of OPA1 restored mitochondrial dynamics with increased mitochondrial network branches and branch length in FGF21 knock down cardiomyocytes or cardiomyocytes under CoCl_2_ stimulation (Fig. 6A–D). Overexpression of OPA1 also restored mitochondrial function and reduced mitophagy induced by FGF21 deficiency or CoCl_2_ stimulation (Fig. 6E–I).

**Fig. 7:**
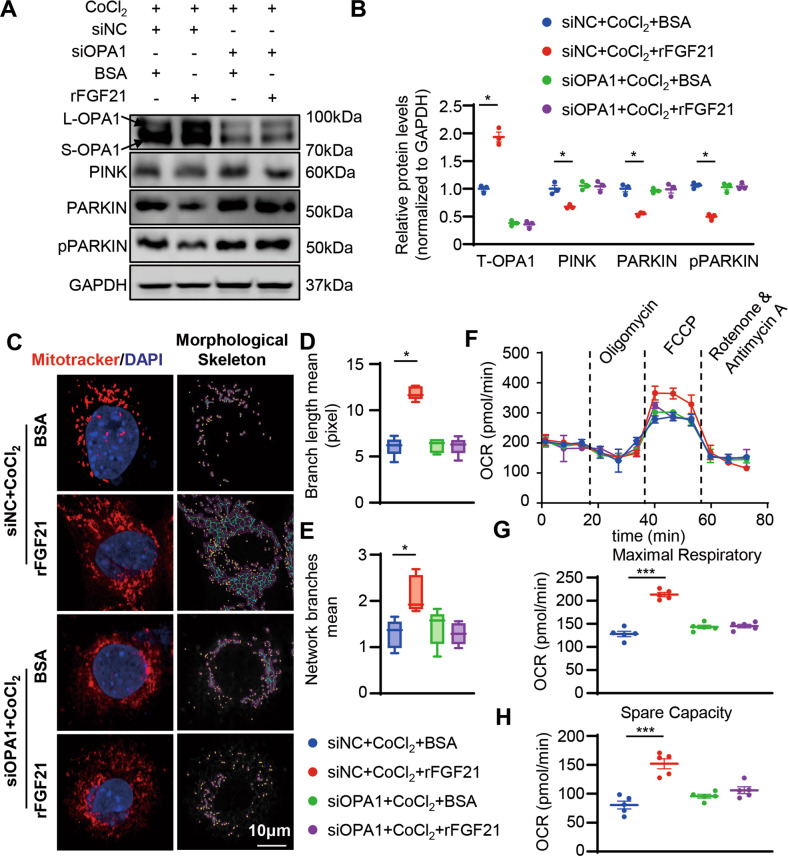
Knocked down OPA1 impair the mitochondrial protection effect of rFGF21. **A**, **B** Representatives immunoblot images and quantification of the OPA1, PINK, PARKIN, and p-PARKIN in siNC or siRNA targeting OPA1 (siOPA1) with or without rFGF21 (50 nM) treated HL-1 cells under CoCl_2_ (200 nm, 24 h). *n* = 3 per group. **C** Representative images of mitochondrial network visualized by Mitotracker staining (MitoTracker in red and DAPI in blue) in siNC or siOPA1 with or without rFGF21 (50 nM) treated HL-1 cells under CoCl_2_ (200 nm, 24 h), and mitochondrial morphological skeleton generated by MiNA (a ImageJ macro tool). **D**, **E** Quantification of mitochondrial network, branch length, and network branches was analyzed by MiNA in ImageJ. Summary statistics for all cells (30 cells from 3 experiments were analyzed, 10 cells per experiment), box plots show median (horizontal lines), first to third quartile (box), and the most extreme values (vertical lines). **F** Representative oxygen consumption curves in siNC or siOPA1 with or without rFGF21 (50 nM) treated HL-1 cells under CoCl_2_ (200 nm, 24 h). Basal respiration rate was measured followed by proton leak after the addition of oligomycin (1.5 μM), maximal respiration was measured after the addition of FCCP (0.5 μM), and non-mitochondrial respiration after the addition of rotenone and antimycin (0.5 μM). **G**, **H** Quantification analysis of maximal respiratory and spare capacity. *n* = 5 per group. Data are expressed as the mean ± SD (**F**) and the mean ± SEM, with individual data points (**B**, **G**, **H**). Data were analyzed by ordinary one-way ANOVA with Tukey’s multiple comparisons test (**B**, **D**, **E**, **G**, **H**).

To further confirm that OPA1 plays an important role in FGF21-induced mitochondrial protection, we used siRNA to ablate OPA1 levels in cardiomyocytes, administered rFGF21 under CoCl_2_ stimulation, and investigated mitophagy activation, mitochondrial morphological change, and mitochondrial function. Ablation of OPA1 compromised the rescuing effect of rFGF21 on inhibiting mitophagy activation induced by CoCl_2_ (Fig. 7A, B). The rescuing effect of rFGF21 on restoring mitochondrial dynamics and function was also compromised (Fig. 7C–H). These results suggested that FGF21 maintains mitochondrial homeostasis by regulating OPA1 levels.

### FGFR1 regulated OPA1 degradation through E3 ligase FBXW11

**Fig. 8:**
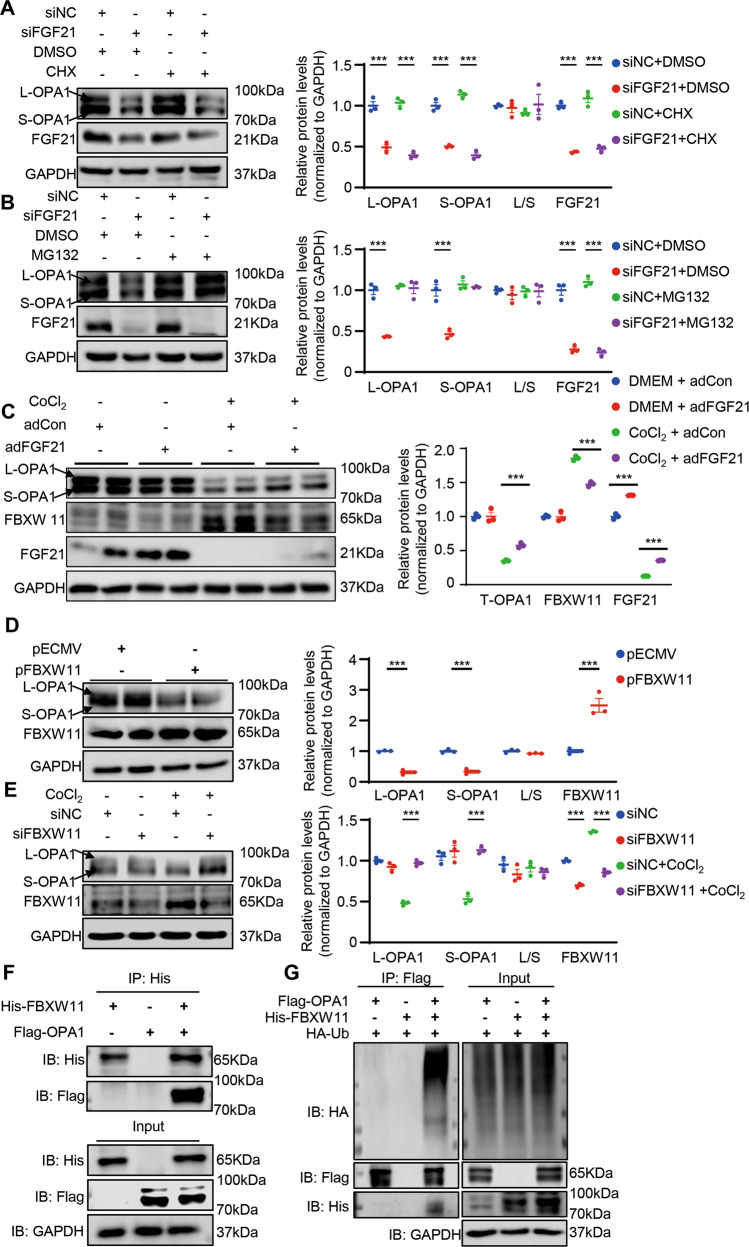
FBXW11 mediated FGF21 regulation of OPA1 degradation. **A** Representatives immunoblot images and quantification of the OPA1, and FGF21 in siNC or siFGF21 with or without CHX (20 μg/ml, 6 h) treated HL-1 cells. *n* = 3 per group. **B** Representatives immunoblot images and quantification of the OPA1, and FGF21 in siNC or siFGF21 with or without MG132 (100 μM, 6 h) treated HL-1 cells. *n* = 3 per group. **C** Representatives immunoblot images and quantification of the OPA1, FBXW11, and FGF21 in adCon or adFGF21 (transfected 24 h) treated HL-1 cells with or without CoCl2 (200 nm, 24 h) stimulation. *n* = 3 per group. **D** Representatives immunoblot images and quantification of the OPA1, and FBXW11 in pECMV or FBXW11 plasmid (pFBXW11) treated HL-1 cells. *n* = 3 per group. **E** Representatives immunoblot images and quantification of the OPA1, and FBXW11 in siNC or siRNA targeting FBXW11 (siFBXW11) treated HL-1 cells. *n* = 3 per group. **F**, **G** IP analysis showed that FBXW11 could interacted with OPA1 and increased OPA1 ubiquitination. Data are expressed as the mean ± SEM, with individual data points. Data were analyzed by ordinary one-way ANOVA with Tukey’s comparisons test (**A**–**C**, **E**), two-tailed unpaired Student’s t test (**D**).

As FGF21 deficiency and overexpression had no effect on the mRNA levels of OPA1, we further tried to determine whether the decrease in OPA1 protein was due to a decrease in synthesis or an increase in degradation. We found that MG132, which is proteosomes inhibitor, inhibited the degradation of OPA1 caused by FGF21 deficiency, suggesting that FGF21 regulates the degradation of OPA1 (Fig. 8A, B). Furthermore, after using the protein synthesis blocker cycloheximide (CHX) to counteract OPA1 synthesis, the decrease in OPA1 was still observed with FGF21 deficiency (Fig. 8A, B). These results suggested that increased degradation was the mechanism underlying OPA1 protein reduction caused by FGF21 deficiency.

To determine the binding of E3 ligase to OPA1, we used UbiBrowser to predict the potential OPA1 E3 ligase (Fig. S11A). We evaluated the expression of several E3 ligases; an increase in FBXW11 protein was detected in the heart of FGF21 KO mice compared to WT, however, there were no differences at the mRNA level (Fig. S11B–D). The predicted ubiquitination sites of OPA1 by FBXW11 were on the N-terminal side of the OMA1 and YME1L cleavage sites, indicating that FBXW11 could bind to both the L-OPA1 and S-OPA1 isoforms. The subcellular localization of OPA1 is mainly in the mitochondria; therefore, the subcellular localization of FBXW11 impacts whether it is a potential E3 ligase that binds to OPA1. To confirm the subcellular localization of FBXW11, we evaluated FBXW11 levels in mitochondrial and cytosolic lysates isolated from neonatal mouse cardiomyocytes. The results showed that FBXW11 was localized in both the mitochondria and the cytosol (Fig. S11E).

With CoCl_2_ stimulation, FBXW11 markedly increased in cardiomyocytes, associated with the reduction of OPA1 and FGF21 (Fig. S12A, B). As expected, silencing of FGF21 increased FBXW11 expression, whereas overexpression of FGF21 decreased FBXW11 expression (Fig. S12C–F, Fig. 8C). Moreover, OPA1 degradation increased after overexpression of FBXW11, and transfection of siRNAs targeting FBXW11 reduced OPA1 degradation in cardiomyocytes caused by CoCl_2_ stimulation (Fig. 8D, E). This further suggested that OPA1 was a possible physiological substrate of FBXW11. To confirm this result, we detected the interaction between FBXW11 and OPA1 using an immunoprecipitation (IP) assay. Overexpression of FBXW11 increased the ubiquitination of OPA1 (Fig. 8F, G).

Because FGF21 exerts its effect mainly through the activation of FGFR1, a tyrosine kinase receptor, we investigated whether FGFR1 could interact with FBXW11. We found increased FBXW11 in transfected siFGFR1 cardiomyocytes (Fig. S13A, B). Following evaluation of FGFR1 phosphorylation levels, cells transfected with a FGFR1 plasmid showed increased FBXW11 degradation (Fig. S13C, D). Collectively, these results supported the notion that OPA1 is a degradation substrate of FBXW11, and that FGFR1 activation could decrease FBXW11 levels.

## Discussion

In the present study, we demonstrated that cardiomyocyte mitochondria are direct targets of autocrine and endocrine FGF21 (summarized in Fig. S15A). The absence of FGF21 is associated with damaged mitochondrial structure and function, both in vitro and in vivo, which is caused by the increased degradation of OPA1. Endogenous or exogenous FGF21 protected mitochondria in cardiomyocytes from stress stimulation, both in vitro and in vivo. We also confirmed that the degradation of OPA1 is mediated by FBXW11, an E3 ligase of OPA1, and the inhibition of OPA1 degradation is mainly mediated by activation of FGFR1 rather than FGFR3.

FGF21 is a key regulator of metabolism, especially lipid and glucose metabolism [29], and plays a role in many mitochondria-related diseases [30–32]. There have also been many reports on cardiovascular benefits [33]. However, the mechanism by which FGF21 exerts beneficial effects on cardiac function remains unclear. Previous studies have found cardioprotective effects of FGF21 from cardiac hypertrophy [6] and in a mouse model of experimental myocardial infarction [8, 34]. There have also been reports that FGF21 could protect against diabetic cardiomyopathy by inhibiting ROS [35, 36]. Although there are reports involving mitochondrial status of FGF21 in muscle [32] and serum [30, 31], there are no reports on whether FGF21 affects cardiomyocyte mitochondrial morphology or function. We observed severely damaged mitochondria in cardiomyocytes of FGF21 KO mice, accompanied by increased mitophagy. Moreover, we found that FGF21 plays a key role in maintaining normal mitochondrial function. In summary, our results are consistent with the fact that the heart is a direct target of FGF21 and plays a key role in maintaining mitochondrial homeostasis.

OPA1 has emerged as a critical regulator of mitochondrial fusion and fission [37, 38], and normal mitochondrial dynamics are important for cardiac metabolism. OPA1 is required for PTP-induced mitochondrial swelling, mitochondrial respiratory supercomplex assembly, and oxidative phosphorylation [39]. In the present study, we found that OPA1 is significantly decreased in FGF21 KO mice, and FGF21 could regulate OPA1 degradation in vitro. However, there are no reports on the association between FGF21 and OPA1. Previous studies have reported that FGF21 can reduce ROS production and protect mitochondria from damage [35]. Our results revealed a new mechanism by which FGF21 regulates mitochondrial homeostasis. We further investigated the degradation of OPA1, finding that FBXW11 degraded OPA1, which was inhibited by FGF21. In an in vitro study, we found that CoCl_2_ stress and FGF21 deficiency both increased FBXW11 levels, which are predicted to be OPA1 E3 ligases. Overexpression of FBXW11 downregulated OPA1 and knockdown of FBXW11 upregulated OPA1. The results of the present study show that FGF21 could regulate OPA1 degradation through FBXW11, which provides more details about FGF21 regulation of OPA1.

It has been established in previous studies that cardiac cells can secret FGF21, although at a lower level than liver or white adipose tissue [40–42]. FGF21, which acts on cardiomyocytes, could originate from the circulation or from the cardiac tissue itself. In our study, we showed that FGF21 could exert its effect through both endocrine and autocrine functions by adding exogenous recombinant FGF21 or by overexpressing adFGF21. Both of these methods had a cardiac protective effect. We then knocked down FGFR1 or FGFR3 to confirm which receptor mediated the cardioprotective effect of FGF21. Knockdown of FGFR1 significantly diminished the protective effects of FGF21, and overexpression increased the activation of FGFR1, inhibited OPA1 degradation, and downregulated FBXW11. These results revealed that the activation of FGFR1 could increase FBXW11 degradation and inhibit OPA1 degradation.

Collectively, the findings reported in this study indicate that FGF21 is required for maintaining mitochondrial homeostasis, and under stress stimulation FGF21 restores mitochondrial damage. This suggests that the FGF21-FGFR1-OPA1 axis could be a potential therapeutic target for cardiac dysfunction.

## Materials and methods

### Animals

FGF21 knockout mice in C57BL/6J background purchased from Cyagen Biosciences (Suzhou, China), which is conventional knockout type. The gRNAs target sequence are gRNA1 (matching forward strand of gene): TGTGTCAAATATCACGCGTCAGG and gRNA2 (matching reverse strand of gene): GAGTGGGTAACCACGATTGTTGG. The genotyping strategy and PCR screening results were showed in Fig. S1A, and the PCR primers for screening are Forward primer (F1): 5’-CAGACCCAGGAGTGTAGACTTCAG-3’, and Reverse primer (R1): 5’-CCAGTGGTTCCATTCTCAGTAC-3’, (R3): 5’-AGCTGAGAAGACACTAAGGCTGTC-3’. The band of FGF21KO homozygotes mice is 906 bp, the band of FGF21KO heterozygotes is 906 bp/624 bp and the band of wild-type mice is 624 bp. All animals were housed in temperature-controlled cages with a 12-h light dark cycle and provided free access to food and water. All animal study procedures were approved by the Cardiovascular Research Institute and the Department of Cardiology of the General Hospital of Northern Theater Command and conformed to the National Institutes of Health (NIH) Guide for the Care and Use of Laboratory Animals. Adeno-associated virus AAV-TNT- FGF21 (1 × 10^11^ vg of adeno-associated virus each mouse) and control virus AAV-TNT ((1 × 10^11^ vg of adeno-associated virus each mouse) from HanbioTech (Shanghai, China) were injected into the tail of mice.

### Glucose tolerance tests and insulin tolerance tests

For glucose tolerance tests, fasting blood glucose levels from tail blood were measured after an overnight fasting, using the glucometer. Mice were then given an intraperitoneal injection of glucose at a dose of 2.0 g per kg body weight. We measured blood glucose at basal, 30, 60, 90, and 120 min from tail blood using the glucometer.

For insulin tolerance tests, fasting blood glucose levels from tail blood were measured after a 4 h of fasting, using the glucometer. Mice were then given an intraperitoneal injection of insulin at a dose of 0.75 units per kg body weight. We measured blood glucose at basal, 30, 60, 90, and 120 min from tail blood using the glucometer.

### Echocardiography measurements

Echocardiography was performed using the Vevo 2100 Ultrasound System (FUJIFILM, Visual Sonics). Mouse cardiac echocardiography measurements were performed under 1% isoflurane anesthesia to detect cardiac function, and mouse cardiac function was detected using a 30-MHz high-frequency scan head as previously described. Parasternal long-axis images were acquired in B-mode with appropriate positioning of the scan head and the maximum LV length identified. In this view, the M-mode cursor was positioned perpendicular to the maximum LV dimension in end-diastole and systole, and M-mode images were obtained for measuring wall thickness and chamber dimensions. Speckle tracking-based strain analyses were performed on parasternal long-axis B-mode loops using the Vevo Strain Software (Vevo LAB) as previously described [43].

### Transmission electron microscopy (TEM)

First, fresh hearts removed from mice or freshly collected cardiomyocytes were perfused with an electron microscope fixing solution (4% paraformaldehyde and 1% glutaraldehyde in 0.1 mol/L sodium cacodylate buffer, pH 7.4, and 4% sucrose). Then, 2% osmium tetroxide and 0.8% potassium ferrocyanide in a 0.1 mol/L sodium cacodylate buffer were employed to pre-fix for 2 h and odium cacodylate buffer was used to wash three times. The samples were dehydrated using a density gradient of alcohol and acetone. Finally, the samples were cut into 60–80 nm ultrathin slices, dyed, and dried overnight at room temperature. The sections were imaged and analyzed using an H-7800 TEM (Hitachi High-Technologies Europe GmbH, Krefeld, Germany). The average number of mitochondria from each sample was determined from a randomly selected pool of 15 to 20 fields under each condition. Mitochondrial cristae abundance and form in TEM images was evaluated by using a five-grade scoring system as previously described [44], assign a score between 0 and 4 based on the quantitative number of cristae and their appearance (0—no sharply defined crista, 1—greater than 50% of the mitochondrial area without cristae, 2—greater than 25% of mitochondrial area without cristae, 3—many cristae (over 75% of area) but irregular, 4—many regular cristae). Number of mitochondrial, average mitochondria cross-sectional area, cristae number were determined as previously described with ImageJ [45].

### Cardiac perfusion

Left ventricular cardiomyocytes are isolated enzymatically using a Langendorff perfusion system as previously described [46]. Briefly, Langendorff perfusion system was set up firstly, then mice were anesthetized, and hearts were transferred to a 60-mm dish and wash it with NT solution. Mount the aorta onto the Langendorff perfusion cannula, and then firmly ligate the aorta onto the cannula. Then switch the perfusate to A solution (Tyrode solution + 10 mM Taurine, 1 mg/mL BSA) to stop the heartbeat and wash the blood. Next, switch the perfusate to E isolation solution (A solution + 0.6 mg/mL type II collagenase) for enzyme digestion for 12 min. Finally, place the heart in a 35-mm dish containing KB solution and cut off the LV tissue into small pieces. Centrifuge at 150 × *g* for 30 s and discard the supernatant. Re-suspend the myocytes in 10 mL KB solution, free settle for 6 min, discard the supernatant, and re-suspend the pellet in KB solution for further treatment.

### Histological and immunofluorescent study

Heart tissues were dissected out and fixed with 4% paraformaldehyde. 5 μm cross-sections from the fixed hearts were stained with Sirius red according to the manufacture’s protocol. For Mitotracker staining, the fresh cardiomyocytes were fixed in 10% formalin for 30 min at room temperature and then washed 3 times in PBS. The fixed sections were then incubated with 4 μM Mitotracker dye (Thermo Fisher Scientific) for 30 min. After washing, sections were stained with 4’,6’-diamidino-2-phenylindole (DAPI, Thermo Fisher Scientific) and covered with glass coverslips. All slides were examined under the Zeiss colocalization microscope. Isolated adult cardiomyocytes with distorted mitochondria are characterized by distorted organization, over splitting, and swelling mitochondrial morphology [21]. Mitochondrial network morphology analysis was performed with ImageJ as previously described [47].

### Cell culture

Primary mouse cardiomyocytes were prepared from neonatal mice (days 0–2), digested with 0.2% collagenase II, neutralized with two times the volume of serum, and filtered with a 100 μm screen. Cardiomyocytes were then cultured in H-DMEM supplemented with 20% FBS, penicillin (100 U/mL), and streptomycin (100 μg/mL) at 37 °C with 5% CO_2_ and 95% air. HL-1 cells were purchased from ATCC (Manassas, VA). The cells were then divided into different groups according to the experiment.

### Mitochondrial extraction

Mitochondrial from neonatal cardiomyocytes were performed by using the Mitochondria Isolation Kit for Cultured Cells (Pierce, Rockford, USA). Mitochondria were extracted by using reagent-based method, followed by centrifugation at 1300 × *g* for 10 min at 4 °C. The supernatant was further centrifuged at 17,000 × *g* for 15 min at 4 °C to pellet the mitochondria. The crude mitochondrial fraction was resuspended for washing and centrifuged at 17,000 × *g* for 15 min at 4 °C. The pellets were collected as the mitochondrial fraction.

### Gene silencing and adenovirus infection

The siRNA transfections in the cardiomyocytes of neonatal mouse and HL-1 cells were performed using RNAiMAX (Thermo Fisher Scientific, USA) as previously described [48]. Specifically, to knockdown FGF21, FGFR1, FGFR3, FBXW11, and OPA1 in cardiomyocytes, cardiomyocytes were transfected with mouse siRNA (120 nmol/l) (RIBOBIO, Guangzhou, China) of the above protein along with the corresponding non-specific control siRNA (120 nmol/l) (RIBOBIO, Guangzhou, China). After 24 h transfection of siRNA, cardiomyocytes were prepared for the following procedure. Detailed siRNA targeting sequences are listed in Supplementary Table S3. For overexpression of FGF21, adenovirus vector encoding FGF21 (OBiO Technology, Shanghai, China) was used to transfect primary neonatal mouse cardiomyocytes and HL-1 cells.

### Reagent treatment

Recombinant mouse FGF21 protein (rFGF21) purchased from Abcam (ab63277), and it was redissolved in 0.5‰ bovine serum albumin (BSA) (Thermo Fisher Scientific) to prepare a stock solution. We use rFGF21 (50 ng/ml) for 24 h in the study, and BSA (50 ng/ml) as control.

### Western blotting

First, protein lysates were centrifuged at 12,000 × *g* at 4 °C for 15 min, prepared with loading buffer, and boiled for 5 min. Then, they were electrophoresed with 10% SDS and transferred onto polyvinylidene fluoride membranes (Merck, Millipore). After blocking with 5% defatted milk for 1 h, membranes were incubated with primary antibodies overnight at 4 °C. Membranes were then incubated with horseradish peroxidase-conjugated secondary antibodies for 2 h at room temperature. The target proteins signal was detected using Amersham Imager 680 (GE, USA). Expression quantification was calculated with ImageJ. Detailed information for primary antibodies are listed in Supplementary Table S1.

### Real-time quantitative PCR

Total mRNA was extracted by the Trizol Reagent method (Thermo Fisher Scientific), and complementary DNA was synthesized from 500 ng total RNA by reverse transcription with Prime Script RT reagent kit (Takara) with random hexamer primers. Quantitative real-time PCR amplification was performed using SYBR Green (Takara), and GAPDH gene expression was used for normalization. Detailed primers are listed in Supplementary Table S2.

### Seahorse XF cell mito stress test

HL-1 cells after treatment were seeded in laminin-coated microplates, and culture for 24–48 h before measure the oxygen consumption rate (OCR). The measure of OCR in the Seahorses biosciences XF96 extracellular flux analyzer (Agilent, USA) following the standard procedures as previously described (reference CD). The mitochondrial oxidation respiratory functions were calculated based on the changes of OCR after adding Oligomycin, Carbonyl cyanide-4 (trifluoromethoxy) phenylhydrazone (FCCP), Rotenone, and Antimycin (Fig. S14A). And the compound concertation of mito stress test are Oligomycin 1.5 μM, FCCP 0.5 μM, Rotenone, and Antimycin 0.5 μM. Oligomycin inhibits ATP synthase and impacts or decreases electron flow through the Electron Transport Chain (ETC), resulting a reduction in mitochondrial respiration or OCR. This decrease in OCR is linked to cellular ATP production. H^+^ (Proton) leak is defined as remaining basal respiration not coupled to ATP production. The FCCP-stimulated OCR can then be used to calculate spare respiratory capacity, defined as the difference between maximal respiration and basal respiration. Rotenone, and Antimycin shuts down mitochondrial respiration and enables the calculation of non-mitochondrial respiration driven by processes outside the mitochondria. All the data were analyzed with the Wave package.

### Statistical analysis

Statistical analyses were performed in GraphPad Prism, version 8.0 (GraphPad Software, CA, USA). Data were represented as the mean ± the standard error of the mean (SEM) or mean ± the standard deviation (SD). When two groups were compared, the statistical significance was analyzed using a two-tailed unpaired Student’s t-test. For multiple group comparisons, ordinary one-way ANOVA, two-way ANOVA, or two-way RM ANOVA with Geisser-Greenhouse’s correction was used, followed by Tukey’s multiple comparisons test. Results were considered statistically significant at *p* value less than 0.05. *p < 0.05, **p < 0.01, ***p < 0.001.

## Supplementary information

Supplemental Information

Full and uncropped western blots

Reproducibility checklist

## Data Availability

The data and material that support the findings of this study are available from the corresponding author upon reasonable request.
