# Supplementary material for: FGF21-FGFR1 controls mitochondrial homeostasis in cardiomyocytes by modulating the degradation of OPA1
Source: Cell Death Dis. 2023 May 8;14(5):311. doi: 10.1038/s41419-023-05842-9 (PMC10167257; doi:10.1038/s41419-023-05842-9)
Supplement: Supplementary file 1 — Supplemental Information [file 41419_2023_5842_MOESM1_ESM.pdf]

Supplement Information

FGF21-FGFR1 controls mitochondrial homeostasis in cardiomyocytes by modulating the degradation of OPA1

Figure S1

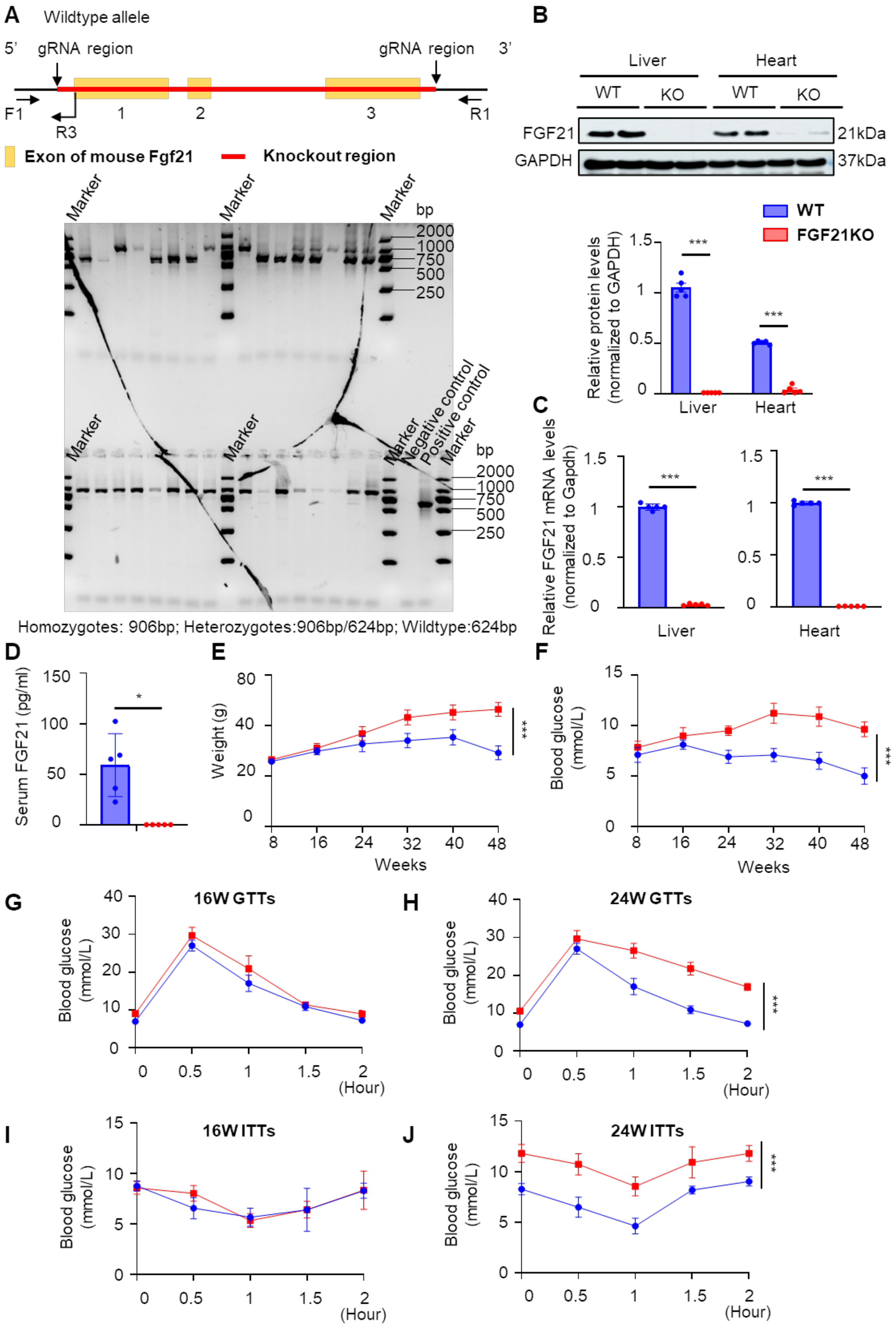
A
Wildtype allele
5'
gRNA region
gRNA region
3'
F1
R3
R1
1
2
3
Exon of mouse Fgf21
Knockout region
Marker
Marker
Marker
bp
2000
1000
750
500
250
Negative control
Positive control
Homozygotes: 906bp; Heterozygotes:906bp/624bp; Wildtype:624bp
B
Liver
Heart
WT
KO
FGF21
GAPDH
21kDa
37kDa
WT
FGF21KO
Relative protein levels (normalized to GAPDH)
C
Relative FGF21 mRNA levels (normalized to Gapdh)
D
Serum FGF21 (pg/ml)
E
Weight (g)
Weeks
F
Blood glucose (mmol/L)
Weeks
G
16W GTTs
H
24W GTTs
I
16W ITTs
J
24W ITTs
Blood glucose (mmol/L)
(Hour)

Figure S1. Deletion of FGF21 does not affect the body weight or glycemic control in young age (before 16W) FGF21 KO mice, but in long-term FGF21 KO mice developed obesity and impaired glycemic control

(A) FGF21 KO mice genotyping strategy and PCR screening result. (B) Representative blots of FGF21 in liver and heart of WT or FGF21 KO mice. n=5 mice. (C) The relative mRNA level of FGF21 in liver and heart of WT or FGF21 KO mice. n=5 mice. (D) Serum FGF21 levels of WT or FGF21 KO mice. n=5 mice. (E) Body weight was measured every 8 weeks in WT and FGF21 KO mice. n=5 mice. (F) Fasting blood glucose was measured every 8 weeks in WT and FGF21 KO mice. n=5 mice. (G-H) 16- and 24-week glucose tolerance tests (GTTs) of WT and FGF21 KO mice. n=5 mice. (I-J) 16- and 24-week insulin tolerance tests (ITTs) of WT and FGF21 KO mice. n=5 mice. Fasting blood glucose were measured after the mice fasted overnight (F). GTTs and ITTs were performed by intraperitoneal injection of 2 g/kg glucose after overnight fasting and 0.75 units/kg insulin after 4 h of fasting, respectively (G-J). Data are expressed as the mean ± SD, with individual data points. Data were analyzed by two-tailed unpaired Student’s t test (B–D, and AUC in G-J) or two-way RM ANOVA with Geisser-Greenhouse’s correction (G-J). (*p< 0.05, **p< 0.01, ***p< 0.001)

Figure S2

A

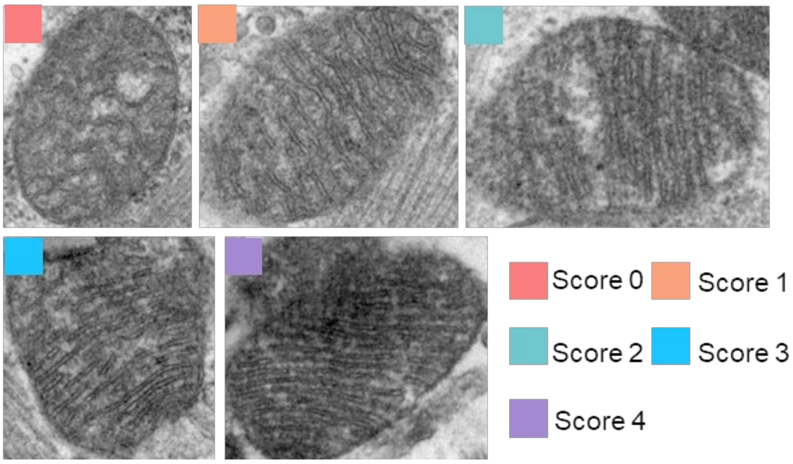

Figure S2. Mitochondrial 5-grade scoring system

(A) Representative mitochondrial appearance in EM using a 5-grade scoring system. Score 4, cristae content >80%, well-defined and intact cristae; score 3, cristae content 60% to 80%, slightly irregular cristae; score 2, cristae content 30% to 60%, major distortions and discontinuous membranes and cristae; score 1, cristae content 10% to 30%, severely fragmented or swollen cristae and warped membranes; score 0, cristae content<10%, severely broken membranes with almost absent cristae.

Figure S3

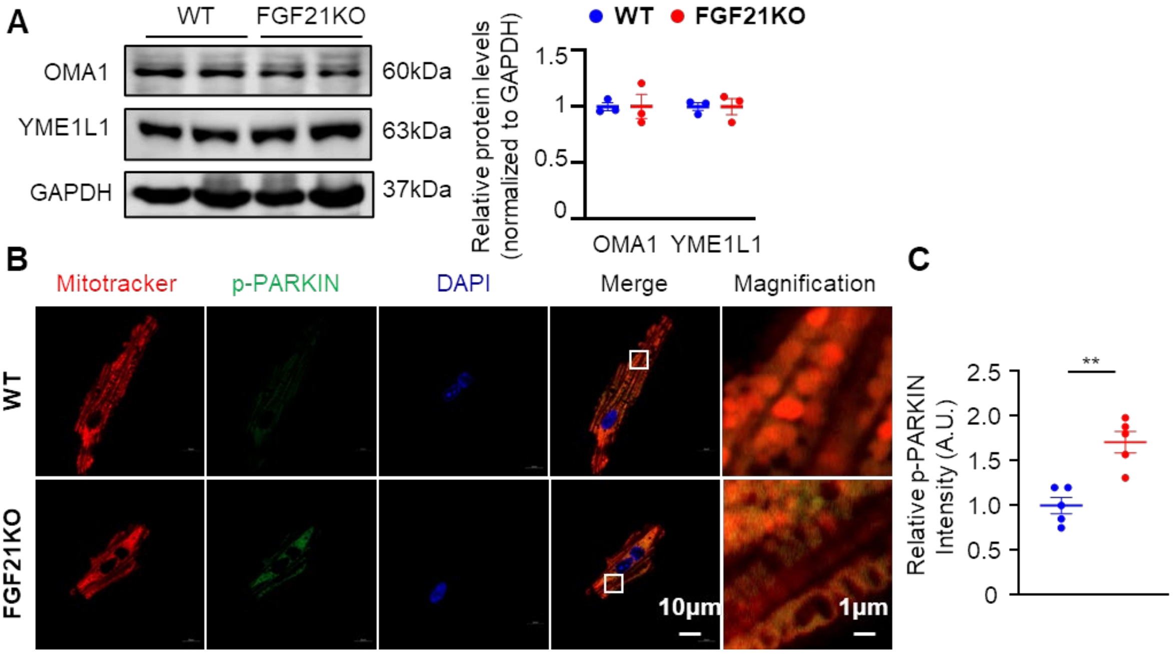
A
WT
FGF21KO
OMA1
YME1L1
GAPDH
60kDa
63kDa
37kDa
WT
FGF21KO
Relative protein levels (normalized to GAPDH)
1.5
1
0.5
0
OMA1
YME1L1
B
Mitotracker
p-PARKIN
DAPI
Merge
Magnification
WT
FGF21KO
10μm
1μm
C
Relative p-PARKIN Intensity (A.U.)
2.5
2.0
1.5
1.0
0.5
0
**

Figure S3. OMA1, YME1L1 levels and p-PARKIN recruitments in FGF21 KO mice heart

(A) Representatives immunoblot images of total proteins extracted from the 16-week-old WT and FGF21 KO mice heart tissues and quantified by densitometric analysis. n = 5 mice. (B-C) Representative confocal images and quantification of the 16-week-old WT and FGF21 KO mice isolated adult cardiomyocytes MitoTracker and p-PARKIN staining. Isolated adult cardiomyocytes (>20) were evaluated of each heart. n = 5 mice. Data are expressed as the mean ± SEM, with individual data points. Data were analyzed by two-tailed unpaired Student's t test (A, C). (* $p < 0.05$, ** $p < 0.01$, *** $p < 0.001$).

Figure S4

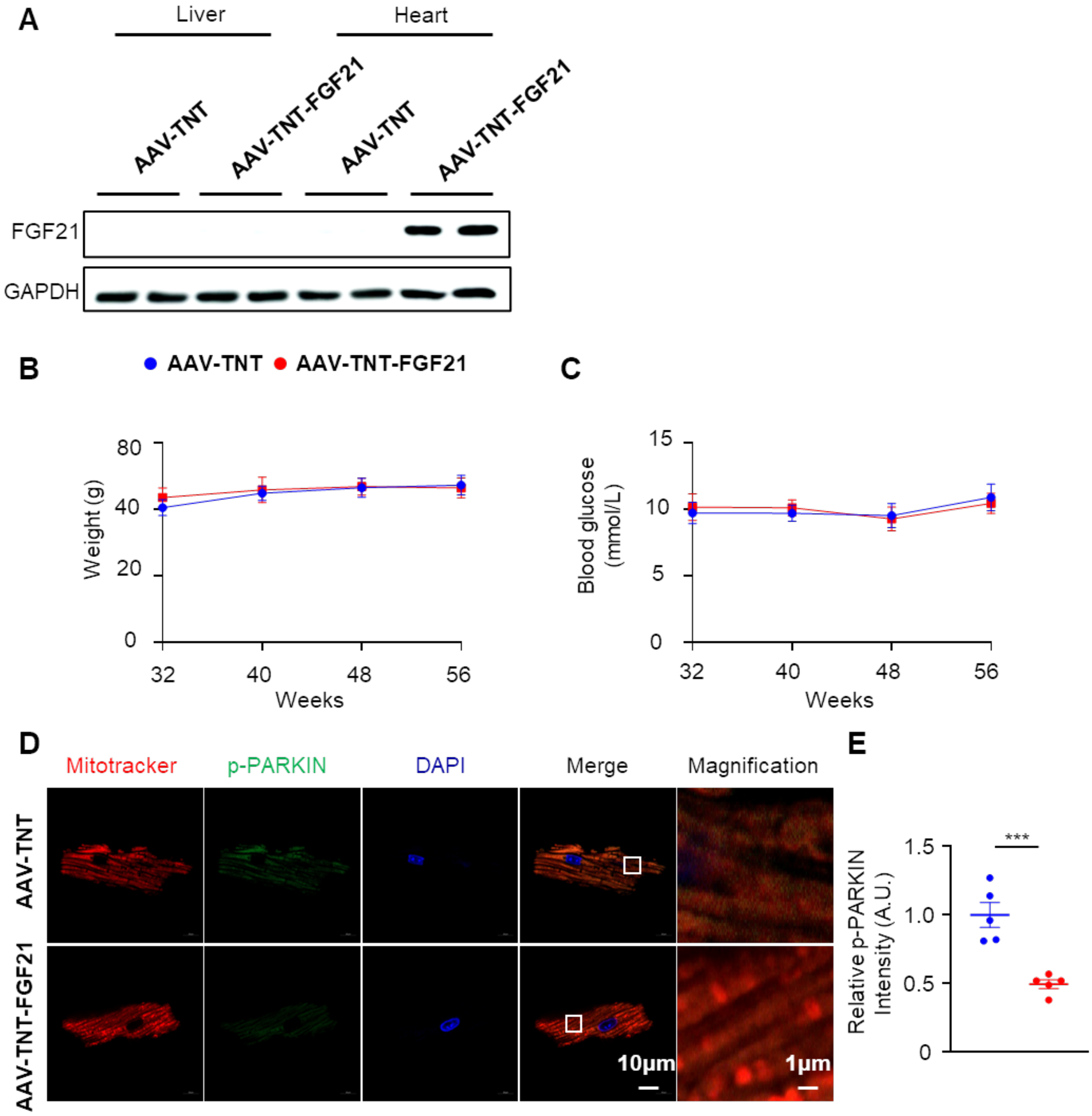

A
Liver
Heart
AAV-TNT
AAV-TNT-FGF21
AAV-TNT
AAV-TNT-FGF21
FGF21
GAPDH
B
AAV-TNT
AAV-TNT-FGF21
Weight (g)
80
40
20
0
32
40
48
56
Weeks
C
Blood glucose (mmol/L)
15
10
5
0
32
40
48
56
Weeks
D
Mitotracker
p-PARKIN
DAPI
Merge
Magnification
AAV-TNT
AAV-TNT-FGF21
10μm
1μm
E
Relative p-PARKIN Intensity (A.U.)
1.5
1.0
0.5
0
***

Figure S4. Cardiac specific overexpression of FGF21 does not affect the body weight or glycemic control.

(A) Representatives immunoblot images of total proteins extracted from the heart and liver tissue of cardiac specific overexpression of FGF21 mice and control. (B) Body weight was measured every 8 weeks in AAV-TNT and AAV-TNT-FGF21 mice. n = 5 mice. (C) Fasting blood glucose was measured every 8 weeks in AAV-TNT and AAV-TNT-FGF21. n = 5 mice. (D, E) Representative confocal images and quantification of the AAV-TNT and AAV-TNT-FGF21 mice isolated adult cardiomyocytes MitoTracker and p-PARKIN staining. Isolated adult cardiomyocytes (>20) were evaluated of each heart. n = 5 mice. Data are expressed as the mean ± SD (B, C) and mean ± SEM, with individual data points (E). Data were analyzed by two-tailed unpaired Student's t test (C, D). (*$p < 0.05$, **$p < 0.01$, ***$p < 0.001$).

Figure S5

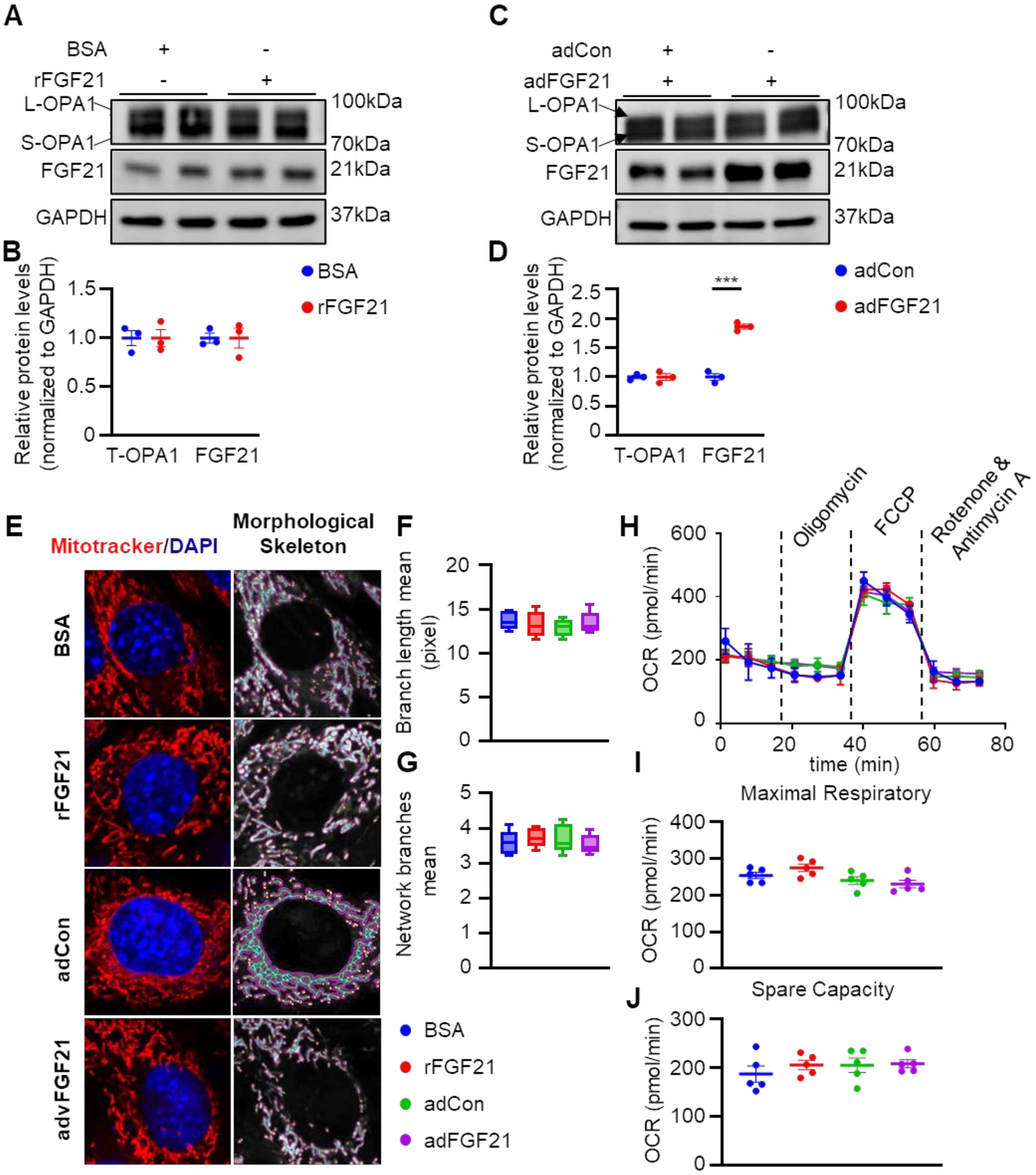

A
BSA + -
rFGF21 - +
L-OPA1
S-OPA1
FGF21
GAPDH
100kDa
70kDa
21kDa
37kDa
B
Relative protein levels (normalized to GAPDH)
BSA
rFGF21
T-OPA1 FGF21
C
adCon + -
adFGF21 + +
L-OPA1
S-OPA1
FGF21
GAPDH
100kDa
70kDa
21kDa
37kDa
D
Relative protein levels (normalized to GAPDH)
***
adCon
adFGF21
T-OPA1 FGF21
E
Mitotracker/DAPI
Morphological Skeleton
BSA
rFGF21
adCon
advFGF21
F
Branch length mean (pixel)
G
Network branches mean
BSA
rFGF21
adCon
adFGF21
H
OCR (pmol/min)
time (min)
Oligomycin
FCCP
Rotenone & Antimycin A
I
Maximal Respiratory
OCR (pmol/min)
J
Spare Capacity
OCR (pmol/min)

Figure S5. Overexpression of FGF21 had no effect on physiological mitochondrial function

(A-D) Representatives immunoblot images and quantification of the OPA1 and FGF21 in neonatal mouse cardiomyocytes treated with rFGF21 (50ng/ml) or adFGF21 (transfected 24h). n = 3. (E-G) Representative images of mitochondrial network visualized by Mitotracker staining (Mitotracker in red and DAPI in blue) in neonatal mouse cardiomyocytes treated with rFGF21 (50ng/ml) or adFGF21 (transfected 24h) and mitochondrial morphological skeleton generated by MiNA (a ImageJ macro tool). (F, G) Quantification of mitochondrial network, branch length and network branches was analyzed by MiNA in ImageJ. Summary statistics for all cells (30 cells from 3 experiments were analyzed, 10 cells per experiment), box plots show median (horizontal lines), first to third quartile (box), and the most extreme values (vertical lines). (H) Representative oxygen consumption curves in HL-1 cells treated with rFGF21 (50ng/ml) or adFGF21 (transfected 24h). Basal respiration rate was measured followed by proton leak after the addition of oligomycin (1.5 μM), maximal respiration was measured after the addition of FCCP (0.5 μM), and non-mitochondrial respiration after the addition of rotenone and antimycin (0.5 μM). (I, J) Quantification analysis of maximal respiratory and spare capacity. n = 5 per group. Data are expressed as the mean ± SEM, with individual data points (B, D, I, J), and the mean ± SD (H). Data were analyzed by two-tailed unpaired Student's t test (A, D), ordinary one-way ANOVA with Tukey's multiple comparisons test (F, G, I, J). (*$p < 0.05$, **$p < 0.01$, ***$p < 0.001$).

Figure S6

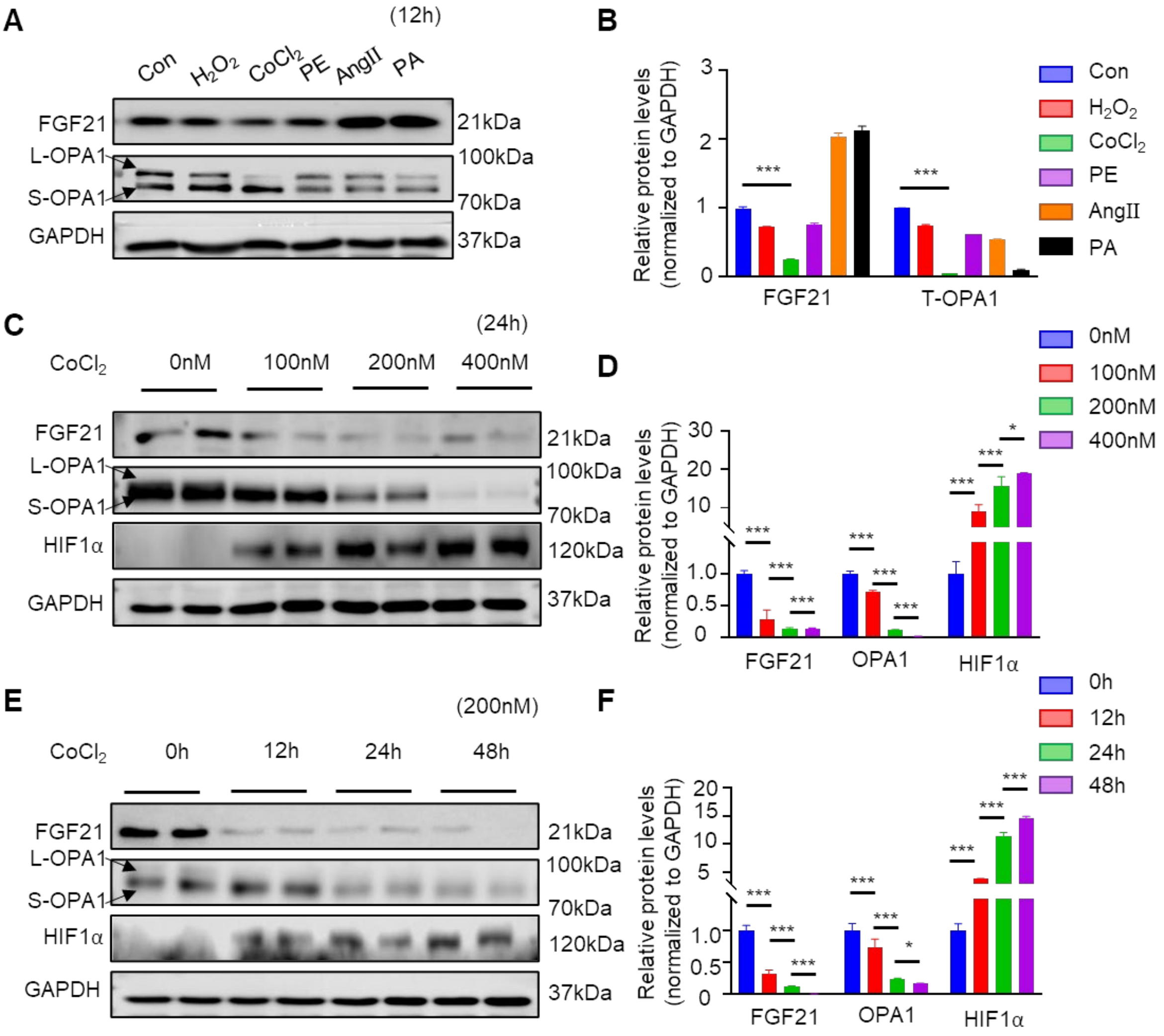

A
(12h)
Con
H2O2
CoCl2
PE
AngII
PA
FGF21
L-OPA1
S-OPA1
GAPDH
21kDa
100kDa
70kDa
37kDa
B
Relative protein levels
(normalized to GAPDH)
3
2
1
0
FGF21
T-OPA1
***
***
Con
H2O2
CoCl2
PE
AngII
PA
C
(24h)
CoCl2
0nM
100nM
200nM
400nM
FGF21
L-OPA1
S-OPA1
HIF1α
GAPDH
21kDa
100kDa
70kDa
120kDa
37kDa
D
Relative protein levels
(normalized to GAPDH)
30
20
10
1.0
0.5
0
FGF21
OPA1
HIF1α
0nM
100nM
200nM
400nM
E
(200nM)
CoCl2
0h
12h
24h
48h
FGF21
L-OPA1
S-OPA1
HIF1α
GAPDH
21kDa
100kDa
70kDa
120kDa
37kDa
F
Relative protein levels
(normalized to GAPDH)
20
15
10
5
1.0
0.5
0
FGF21
OPA1
HIF1α
0h
12h
24h
48h

Figure S6. $CoCl_2$ is a pathological stimulator to induce FGF21 and OPA1 decreasing.

(A, B) Representatives immunoblot images and quantification of the OPA1 and FGF21 in H_2O_2 (300nM), $CoCl_2$ (200nM), PE (100μM), Ang II (1μM), and PA (200mM) treated 24h HL-1 cells. (C, D) Representatives immunoblot images and quantification of the OPA1 and FGF21 in different $CoCl_2$ concentrations (100nM, 200nM, 400nM) treated 24h HL-1 cells. (E) Representatives immunoblot images and quantification of the OPA1 and FGF21 in different $CoCl_2$ (200nM) treatment interval (12h, 24h, 48h) treated HL-1 cells. Data are expressed as the mean ± SD (B, D, F). Data were analyzed by ordinary one-way ANOVA with Tukey's multiple comparisons test (B, D, F). (*p< 0.05, **p< 0.01, ***p< 0.001).

Figure S7

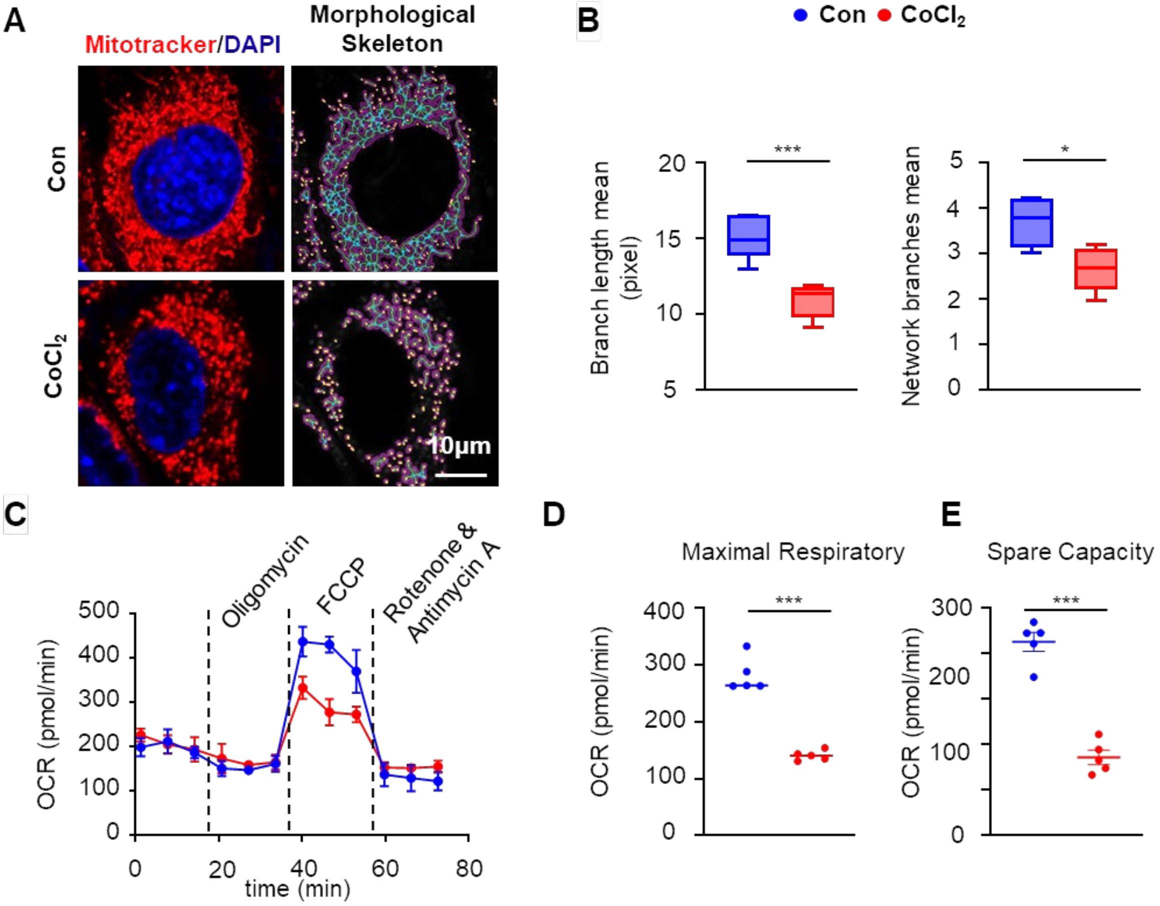

A
Mitotracker/DAPI
Morphological Skeleton
Con
CoCl2
10μm
B
Con CoCl2
Branch length mean (pixel)
Network branches mean
***
*
C
Oligomycin
FCCP
Rotenone & Antimycin A
OCR (pmol/min)
time (min)
D
Maximal Respiratory
E
Spare Capacity

Figure S7. $CoCl_2$ stimulation could cause OPA1 degradation and impair mitochondrial structure and function.

(A) Representative images of mitochondrial network visualized by Mitotracker staining (Mitotracker in red and DAPI in blue) in HL-1 cells treated with $CoCl_2$ (200nM, 24h) and mitochondrial morphological skeleton generated by MiNA (a ImageJ macro tool). (B) Quantification of mitochondrial network, branch length and network branches was analyzed by MiNA in ImageJ. Summary statistics for all cells (30 cells from 3 experiments were analyzed, 10 cells per experiment), box plots show median (horizontal lines), first to third quartile (box), and the most extreme values (vertical lines). (C) Representative oxygen consumption curves in HL-1 cells treated with $CoCl_2$ (200nM, 24h). Basal respiration rate was measured followed by proton leak after the addition of oligomycin (1.5 μM), maximal respiration was measured after the addition of FCCP (0.5 μM), and non-mitochondrial respiration after the addition of rotenone and antimycin (0.5 μM). (D, E) Quantification analysis of maximal respiratory and spare capacity. n = 5 per group. Data are expressed as the mean ± SD (C) and mean ± SEM, with individual data points (D, E). Data were analyzed by two-tailed unpaired Student's t test (A, D), ordinary one-way ANOVA with Tukey's multiple comparisons test (B, D, E). (*p< 0.05, **p< 0.01, ***p< 0.001).

Figure S8

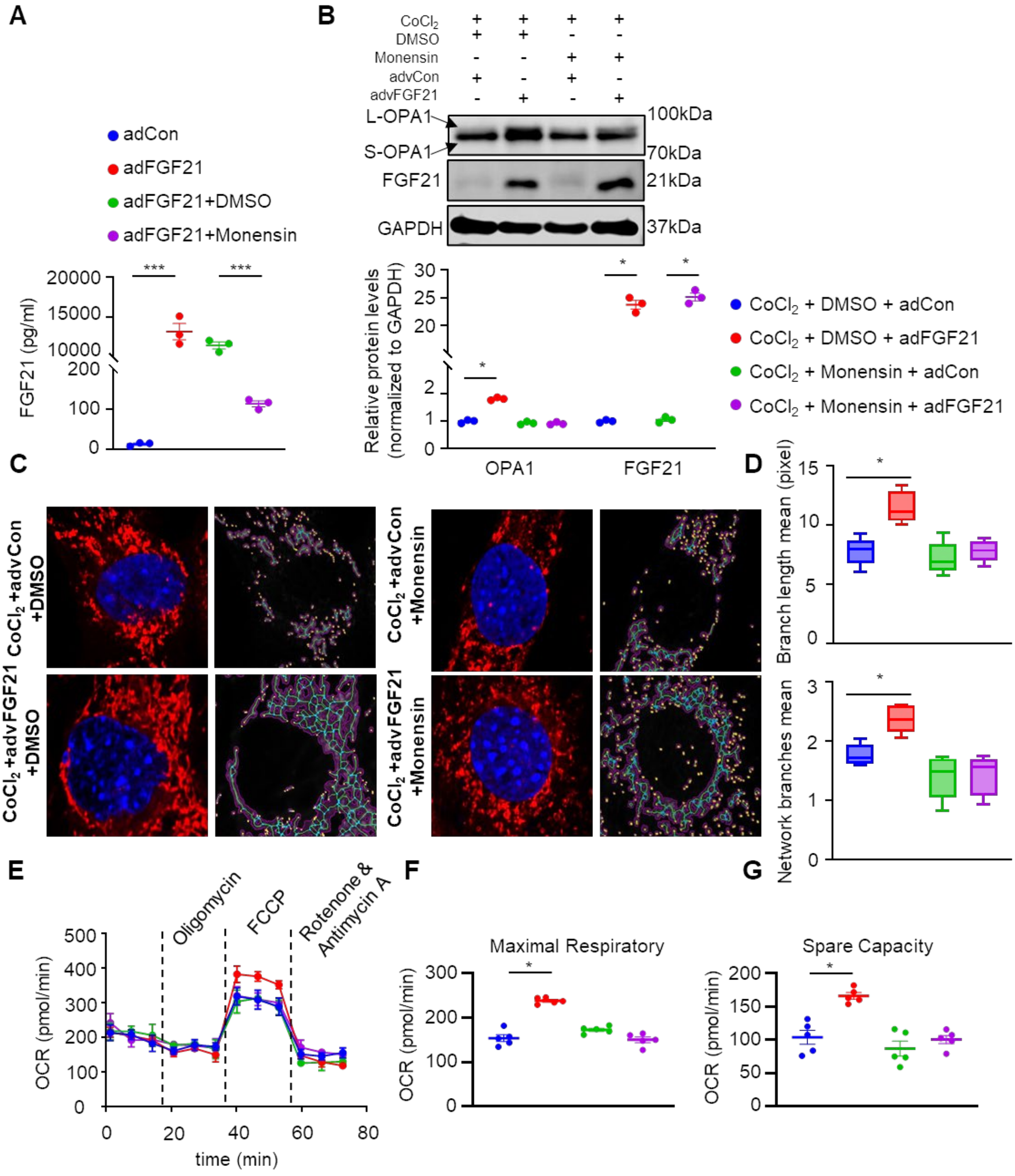
A
B
adCon
adFGF21
adFGF21+DMSO
adFGF21+Monensin
FGF21 (pg/ml)
***
CoCl2
DMSO
Monensin
advCon
advFGF21
L-OPA1
S-OPA1
FGF21
GAPDH
100kDa
70kDa
21kDa
37kDa
Relative protein levels
(normalized to GAPDH)
OPA1
FGF21
CoCl2 + DMSO + adCon
CoCl2 + DMSO + adFGF21
CoCl2 + Monensin + adCon
CoCl2 + Monensin + adFGF21
C
D
CoCl2 +advCon +DMSO
CoCl2 +advFGF21 +DMSO
CoCl2 +advCon +Monensin
CoCl2 +advFGF21 +Monensin
Branch length mean (pixel)
Network branches mean
E
F
G
Oligomycin
FCCP
Rotenone &
Antimycin A
OCR (pmol/min)
time (min)
Maximal Respiratory
Spare Capacity

Figure. S8 Monensin inhibited the protecting effect of adFGF21 on mitochondrial damage induced by $CoCl_2$.

(A) Cell culture supernatants FGF21 levels detected by ELISA, in adFGF21 (transfected 24h) with or without Monensin (5μM, 12h) treated neonatal mouse cardiomyocytes. n = 3. (B) Representatives immunoblot images and quantification of the OPA1 and FGF21 in adFGF21 (transfected 24h) with or without Monensin (5μM, 12h) treated neonatal mouse cardiomyocytes under $CoCl_2$ (200nM, 24h) stimulation. (C) Representative images of mitochondrial network visualized by Mitotracker staining (MitoTracker in red and DAPI in blue) in adFGF21 (transfected 24h) with or without Monensin (5μM, 12h) treated HL-1 cells under $CoCl_2$ (200nM, 24h) and mitochondrial morphological skeleton generated by MiNA (a ImageJ macro tool). (D) Quantification of mitochondrial network, branch length and network branches was analyzed by MiNA in ImageJ. Summary statistics for all cells (30 cells from 3 experiments were analyzed, 10 cells per experiment), box plots show median (horizontal lines), first to third quartile (box), and the most extreme values (vertical lines). (E) Representative oxygen consumption curves in adFGF21 (transfected 24h) with or without Monensin (5μM, 12h) treated HL-1 cells under $CoCl_2$ (200nM, 24h). Basal respiration rate was measured followed by proton leak after the addition of oligomycin (1.5 μM), maximal respiration was measured after the addition of FCCP (0.5 μM), and non-mitochondrial respiration after the addition of rotenone and antimycin (0.5 μM). (F, G) Quantification analysis of maximal respiratory and spare capacity. n = 5 per group. Data are expressed as the mean ± SD (E) and the mean ± SEM, with individual data points (A, B, F, G). Data were analyzed by ordinary one-way ANOVA with Tukey's multiple comparisons test (A, B, D, F, G). (* $p < 0.05$, ** $p < 0.01$, *** $p < 0.001$).

Figure S9

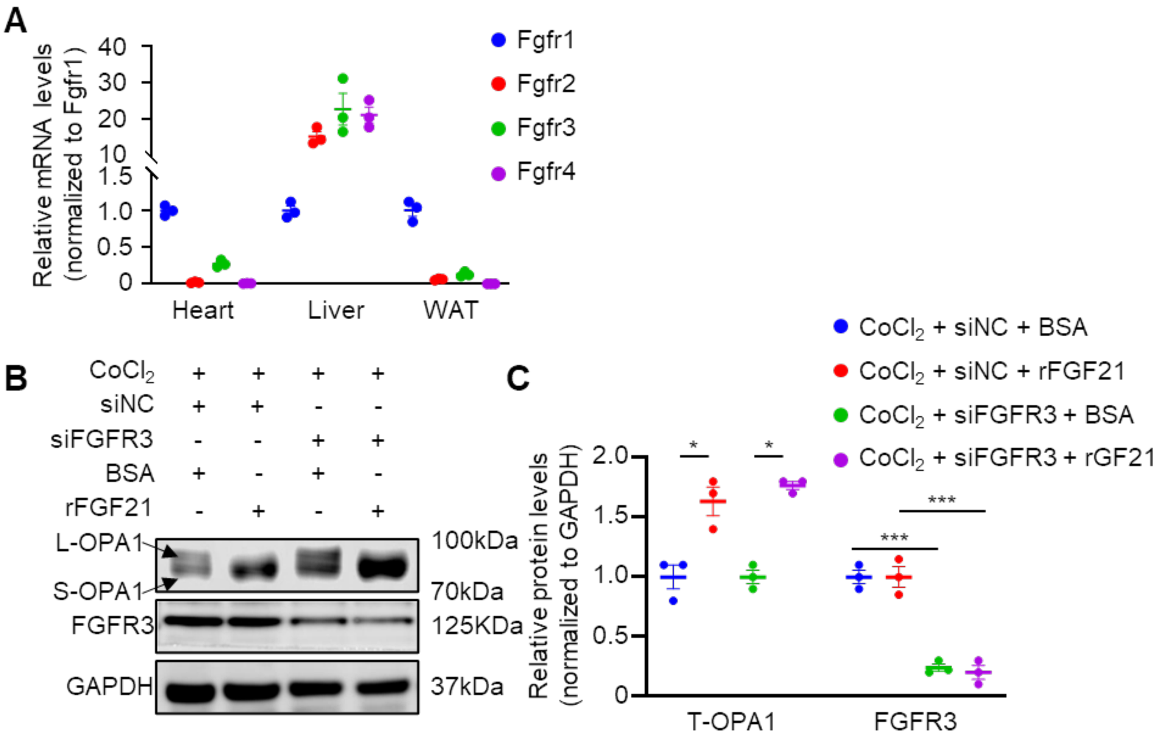
A
Relative mRNA levels (normalized to Fgfr1)
Fgfr1
Fgfr2
Fgfr3
Fgfr4
Heart
Liver
WAT
B
CoCl2
siNC
siFGFR3
BSA
rFGF21
L-OPA1
S-OPA1
FGFR3
GAPDH
100kDa
70kDa
125KDa
37kDa
C
CoCl2 + siNC + BSA
CoCl2 + siNC + rFGF21
CoCl2 + siFGFR3 + BSA
CoCl2 + siFGFR3 + rGF21
Relative protein levels (normalized to GAPDH)
T-OPA1
FGFR3

Figure S9. Knocked down FGFR3 has no effect on inhibition of OPA1 decreasing caused by FGF21.

(A)Relative mRNA levels of Fgfr1-4 normalized to Fgfr1 in mice heart, liver, and white adipose tissue. (B, C) Representatives immunoblot images and quantification of the OPA1 and FGFR3 in siNC or siRNA targeting Fgfr1 (siFGFR3) with or without rFGF21 (50nM) treated neonatal mouse cardiomyocytes under $CoCl_2$ (200nm, 24h). Data are expressed as the mean ± SEM, with individual data points (A, C). Data were analyzed by ordinary one-way ANOVA with Tukey's multiple comparisons test (A, C). (*$p < 0.05$, **$p < 0.01$, ***$p < 0.001$).

Figure S10

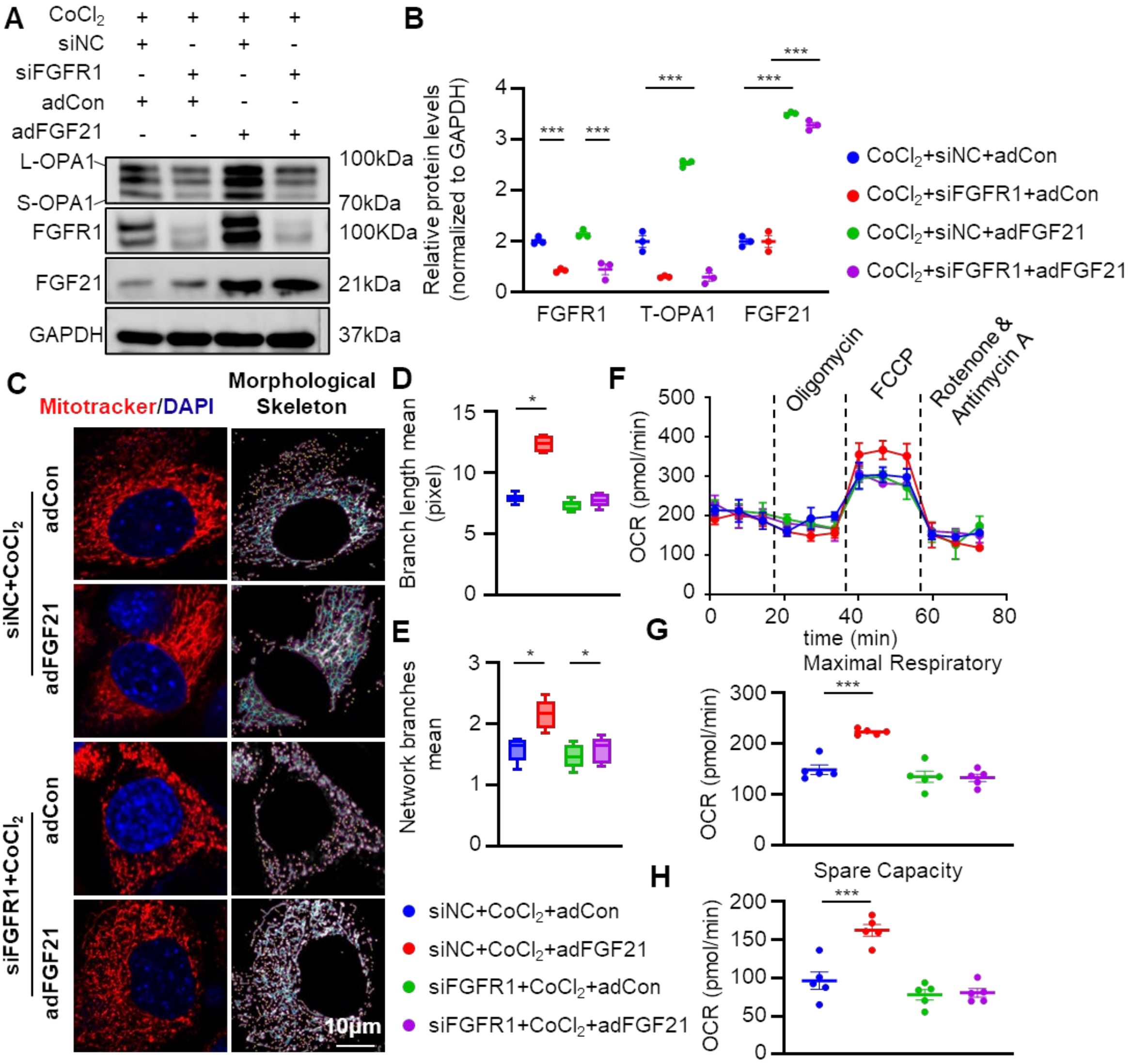

A
CoCl2 + + + +
siNC + - + -
siFGFR1 - + - +
adCon + + - -
adFGF21 - - + +
L-OPA1
S-OPA1
FGFR1
FGF21
GAPDH
100kDa
70kDa
100KDa
21kDa
37kDa
B
Relative protein levels (normalized to GAPDH)
FGFR1
T-OPA1
FGF21
CoCl2+siNC+adCon
CoCl2+siFGFR1+adCon
CoCl2+siNC+adFGF21
CoCl2+siFGFR1+adFGF21
C
Mitotracker/DAPI
Morphological Skeleton
siNC+CoCl2
siFGFR1+CoCl2
adCon
adFGF21
10μm
D
Branch length mean (pixel)
E
Network branches mean
siNC+CoCl2+adCon
siNC+CoCl2+adFGF21
siFGFR1+CoCl2+adCon
siFGFR1+CoCl2+adFGF21
F
OCR (pmol/min)
time (min)
Oligomycin
FCCP
Rotenone & Antimycin A
G
Maximal Respiratory
OCR (pmol/min)
H
Spare Capacity
OCR (pmol/min)

Figure S10. Knocked down FGFR1 impair the adFGF21 mitochondria protection cause by $CoCl_2$.

(A, B) Representatives immunoblot images and quantification of the OPA1 in siNC or siRNA targeting Fgfr1 (siFGFR1) with or without adFGF21 (transfected 24h) treated neonatal mouse cardiomyocytes under $CoCl_2$ (200nm, 24h). (C) Representative images of mitochondrial network visualized by Mitotracker staining (MitoTracker in red and DAPI in blue) in siNC or siFGFR1 with or without adFGF21 (transfected 24h) treated HL-1 cells under $CoCl_2$ (200nm, 24h), and mitochondrial morphological skeleton generated by MiNA (a ImageJ macro tool). (D, E) Quantification of mitochondrial network, branch length and network branches was analyzed by MiNA in ImageJ. Summary statistics for all cells (30 cells from 3 experiments were analyzed, 10 cells per experiment), box plots show median (horizontal lines), first to third quartile (box), and the most extreme values (vertical lines). (F) Representative oxygen consumption curves in siNC or siFGFR1 with or without adFGF21 (transfected 24h) treated HL-1 cells under $CoCl_2$ (200nm, 24h). Basal respiration rate was measured followed by proton leak after the addition of oligomycin (1.5 μM), maximal respiration was measured after the addition of FCCP (0.5 μM), and non-mitochondrial respiration after the addition of rotenone and antimycin (0.5 μM). (G, H) Quantification analysis of maximal respiratory and spare capacity. n = 5 per group. Data are expressed as the mean ± SD (F) and the mean ± SEM, with individual data points (B, G, H). Data were analyzed by ordinary one-way ANOVA with Tukey's multiple comparisons test (B, D, E, G, H). (*$p < 0.05$, **$p < 0.01$, ***$p < 0.001$).

Figure S11

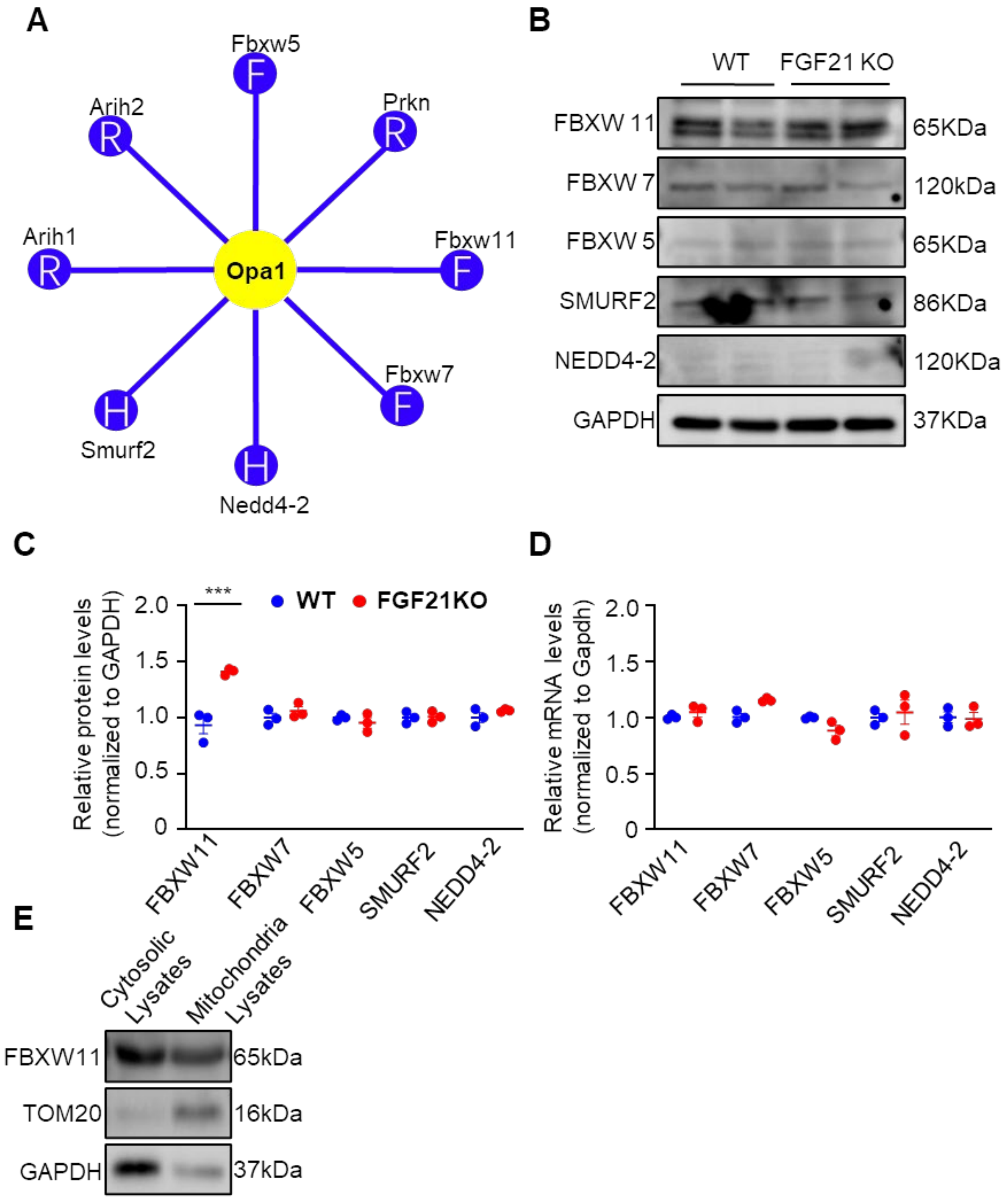

A
Fbxw5
Arih2
Prkn
Arih1
Opa1
Fbxw11
Fbxw7
Smurf2
Nedd4-2
B
WT
FGF21 KO
FBXW 11
65KDa
FBXW 7
120kDa
FBXW 5
65KDa
SMURF2
86KDa
NEDD4-2
120KDa
GAPDH
37KDa
C
***
WT
FGF21KO
Relative protein levels
(normalized to GAPDH)
FBXW11
FBXW7
FBXW5
SMURF2
NEDD4-2
D
Relative mRNA levels
(normalized to Gapdh)
FBXW11
FBXW7
FBXW5
SMURF2
NEDD4-2
E
Cytosolic Lysates
Mitochondria Lysates
FBXW11
65kDa
TOM20
16kDa
GAPDH
37kDa

Figure. S11 Predicted potential OPA1 E3 ligase.

(A) Predict potential OPA1 E3 ligase by using UbiBrowser (http://ubibrowser.bio-it.cn/ubibrowser_v3/). (B, C) Representatives immunoblot images of total proteins extracted from the 16-week WT and FGF21 KO mice heart tissues and quantified by densitometric analysis. n = 3 mice. (D) Relative mRNA levels of the predicted potential OPA1 E3 ligase proteins in the heart of 16-week WT and FGF21 KO mice. n = 3 mice. (E) Representatives immunoblot images of the FBXW11 in cytosolic and mitochondrial lysates isolated from neonatal mouse cardiomyocytes. Data are expressed as the mean ± SEM, with individual data points (C, D). Data were analyzed by two-tailed unpaired Student's t test (C, D). (*$p < 0.05$, **$p < 0.01$, ***$p < 0.001$).

Figure S12

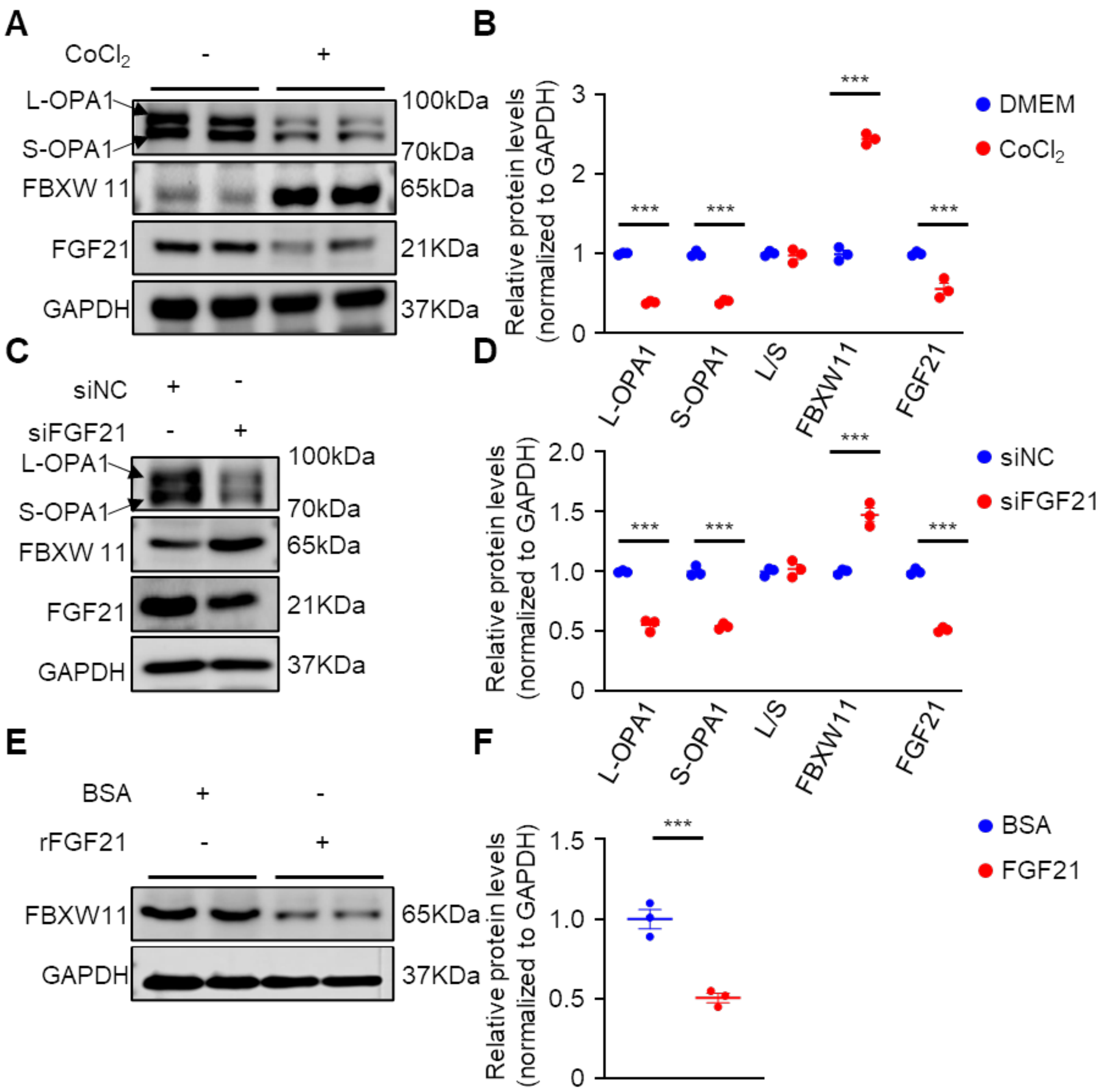
A
CoCl2 - +
L-OPA1
S-OPA1
FBXW 11
FGF21
GAPDH
100kDa
70kDa
65kDa
21KDa
37KDa
B
Relative protein levels (normalized to GAPDH)
DMEM
CoCl2
L-OPA1
S-OPA1
L/S
FBXW11
FGF21
C
siNC + -
siFGF21 - +
D
siNC
siFGF21
E
BSA + -
rFGF21 - +
FBXW11
GAPDH
65KDa
37KDa
F
BSA
FGF21

Figure S12. $CoCl_2$ stimulation and FGF21 deficiency cause increased FBXW11 levels and decreased OPA1 levels

(A, B) Representatives immunoblot images and quantification of the OPA1, FBXW11, and FGF21 in HL-1 cells treated with or without $CoCl_2$ (200nm, 24h) stimulation. (C, D) Representatives immunoblot images and quantification of the OPA1, FBXW11, and FGF21 in siNC or siFGF21 treated HL-1 cells. (E, F) Representatives immunoblot images and quantification of the FBXW11 in rFGF21 (50ng/ml) treated HL-1 cells. Data are expressed as the mean ± SEM, with individual data points. Data were analyzed by two-tailed unpaired Student's t test (B, D, F)

Figure S13

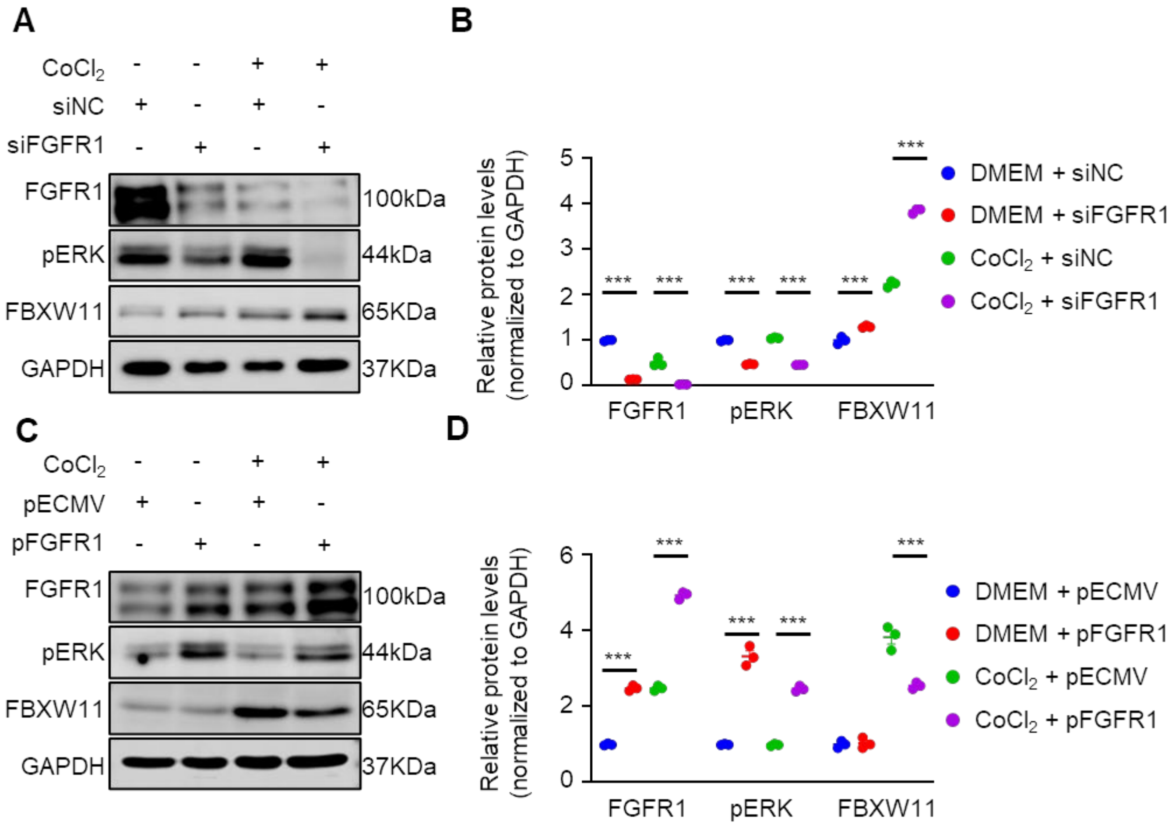
A
CoCl2
siNC
siFGFR1
FGFR1
pERK
FBXW11
GAPDH
100kDa
44kDa
65KDa
37KDa
B
Relative protein levels (normalized to GAPDH)
FGFR1
pERK
FBXW11
DMEM + siNC
DMEM + siFGFR1
CoCl2 + siNC
CoCl2 + siFGFR1
C
CoCl2
pECMV
pFGFR1
FGFR1
pERK
FBXW11
GAPDH
100kDa
44kDa
65KDa
37KDa
D
Relative protein levels (normalized to GAPDH)
FGFR1
pERK
FBXW11
DMEM + pECMV
DMEM + pFGFR1
CoCl2 + pECMV
CoCl2 + pFGFR1

Figure S13. FGFR1 regulates E3 ligase FBXW11

(A, B) Representatives immunoblot images and quantification of the FGFR1, p-ERK, and FBXW11 in siNC or siFGFR1 with or without $CoCl_2$ (200nm, 24h) treated HL-1 cells. (C, D) Representatives immunoblot images and quantification of the FGFR1, p-ERK, and FBXW11 in pECMV or FGFR1 plasmid (pFGFR1) with or without $CoCl_2$ (200nm, 24h) treated HL-1 cells. Data are expressed as the mean ± SEM, with individual data points (B, D). Data were analyzed by ordinary one-way ANOVA with Tukey's multiple comparisons test (B, D). (*$p < 0.05$, **$p < 0.01$, ***$p < 0.001$).

Figure S14

A

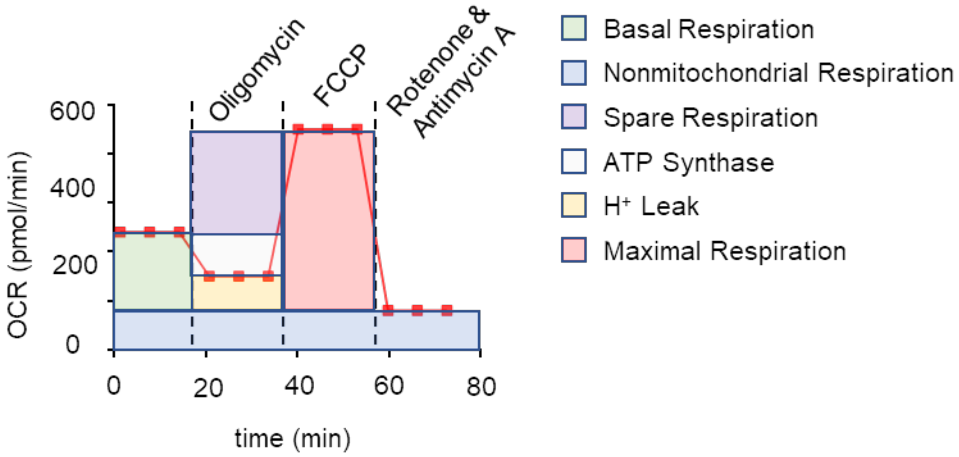

Oligomycin
FCCP
Rotenone &
Antimycin A
600
400
200
0
OCR (pmol/min)
0
20
40
60
80
time (min)
Basal Respiration
Nonmitochondrial Respiration
Spare Respiration
ATP Synthase
H+ Leak
Maximal Respiration

Figure S14. Mitochondrial function analysis

(A) Scheme for measuring variable mitochondrial respiration functions using OCR.

Figure S15

A

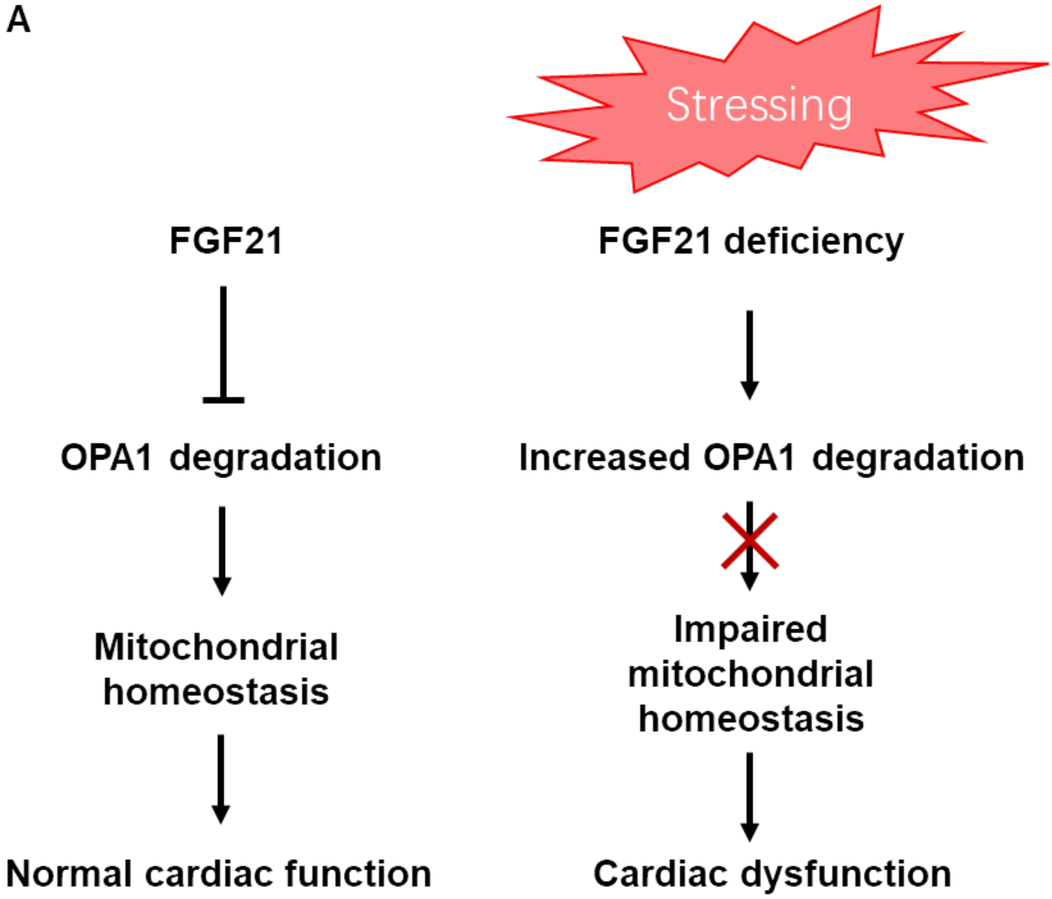
Stressing
FGF21
FGF21 deficiency
OPA1 degradation
Increased OPA1 degradation
Mitochondrial homeostasis
Impaired mitochondrial homeostasis
Normal cardiac function
Cardiac dysfunction

Figure S15.

(A) Schematic diagram showing how FGF21 regulates cardiomyocytes mitochondrial homeostasis.

Table S1

Primary antibodies involved in the study

Antibodies	Company	Catalog number
FGF21	Abcam	ab171941
GAPDH	Cell Signaling Technology	#5174
OPA1	Cell Signaling Technology	#80471
MFN1	Abcam	ab221661
MFN2	Cell Signaling Technology	#9482
DRP1	Cell Signaling Technology	#8570
pDRP1(Ser616)	Cell Signaling Technology	#3455
PINK	Abcam	ab216144
PARKIN	Cell Signaling Technology	#2132
pPARKIN(Ser65)	Cell Signaling Technology	#36866
PGC1α	Cell Signaling Technology	#2178
TOM20	Abcam	Ab186735
HIF1α	Santa Cruz Biotechnology	sc-13515
FGFR3	Cell Signaling Technology	#4574
FGFR1	Cell Signaling Technology	#9740
FBXW11	Affinity Biosciences	DF13009
FBXW7	Affinity Biosciences	DF12400
FBXW5	Affinity Biosciences	DF13011
SMURF2	Affinity Biosciences	DF7683
NEDD4-2	Affinity Biosciences	DF7724
His-Tag	Cell Signaling Technology	#12699
Flag-Tag	Cell Signaling Technology	#2368
HA-Tag	Cell Signaling Technology	#3724
pERK1/2	Cell Signaling Technology	#4370

Table S2

Primers involved in the study

Gene	Forward primers (5'-3')	Reverse primers (5'-3')
m-Gapdh	AGGTCGGTGTGAACGGATTTG	TGTAGACCATGTAGTTGAGGTCA
m-Fgf21	AGGACTCCCCAAACCAGGAT	AGAGTCAGGACGCATAGCTG
m-Bnp	TGGGAGGTCACTCCTATCCT	GGCCATTTCCTCCGACTTT
m-Myh7	AAGGTGAAGGCCTACAAGCG	TGGAGAGGTTATTCCTCGTCG
m-Periostin	GAACGAATCATTACAGGTCCTG	CCTTGGAGACCTCTTTTTGCAAGA
m-Timp1	CTGCTCAGCAAAGAGCTTTC	CTCCAGTTTGCAAGGGATAG
m-Opa1	TCAAGAAAAACTTGATGCTTTCA	GCAGAGCTGATTATGAGTACGATT
m-Mfn1	TGTTTTGGTCGCAAACTCTG	CTGTCTGCGTACGTCTTCCA
m-Mfn2	AGAACTGGACCCGGTTACCA	CACTTCGCTGATACCCCTGA
m-Drp1	ATGCCAGCAAGTCCACAGAA	TGTTCTCGGGCAGACAGTTT
m-Pgc1α	TATGGAGTGACATAGAGTGTGCT	CCACTTCAATCCACCCAGAAAG
m-Fgfr1	TAATACCACCGACAAGGAAATGG	TGATGGGAGAGTCCGATAGAGT
m-Fgfr2	GCAGAGCATCAACTGGCTG	GGTCACGCAAGCGTAGAGG
m-Fgfr3	GGAAAGTGTGGTACCCTCCG	GCCACCAGACCTGTACCATC
m-Fgfr4	TACTGGACACACCCCAACGCAT	GTACACCTTGCAGAGTAGCTCCA
m-Smurf2	AAACAGTTGCTTGGGAAGTCA	TGCTCAACACAGAAGGTATGGT
m-Nedd4-2	GAGTCAAGGGGTTTTTGAGGTT	TGGGAAGCTGAGTCGTTGGA
m-Fbxw5	GAGTTCCGGCGGCTCTATG	AGATGGTCAGGTCGTTGTTCC
m-Fbxw7	GACAGATGAATCGCGTGGTTG	CCACTTGTCCTTGCTGGGTAT
m-Fbxw11	CAAGGTCTTTGTGGCTAGGCTG	TGACGTTCCATTACTGATCTGGAG

Table S3

Small interfering RNA targeting sequences involved in the study

Gene	Company	Targeting sequence
m-Fgf21	RIBOBIO	GAGGTACCTCTACACAGAT
m-Opa1	RIBOBIO	GCTTACATGCAGAATCCTA
m-Fgfr1	RIBOBIO	GGAAATGGAGGTGCTTCAT
m-Fgfr3	RIBOBIO	GCTATTGGCATCGACAAGG
m-Fbxw11	RIBOBIO	GGCTGCAGTTTGATGAGTT
