# Supplementary material for: FGF21-FGFR1 controls mitochondrial homeostasis in cardiomyocytes by modulating the degradation of OPA1
Source: Cell Death Dis. 2023 May 8;14(5):311. doi: 10.1038/s41419-023-05842-9 (PMC10167257; doi:10.1038/s41419-023-05842-9)

Figure 2E

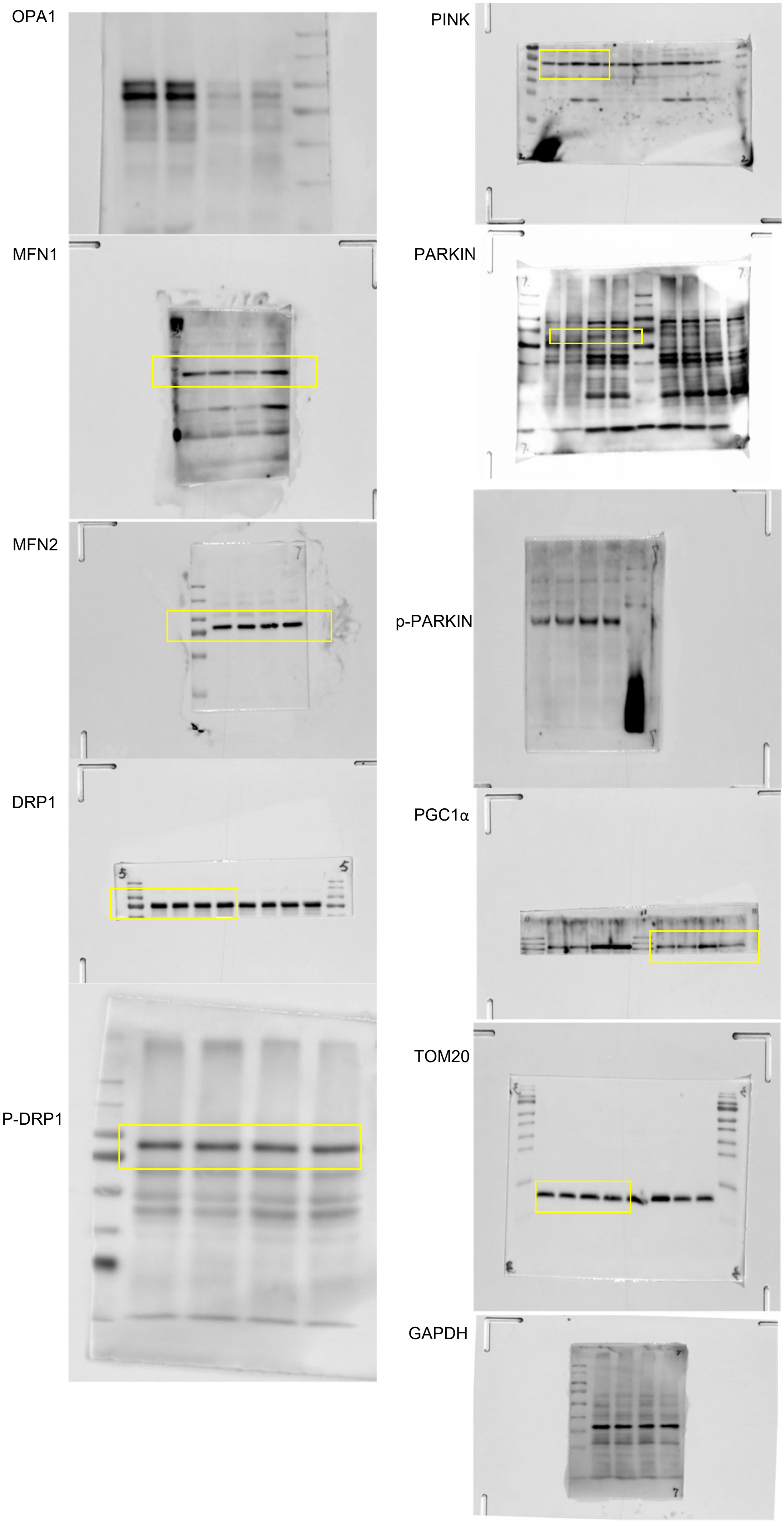
OPA1
PINK
MFN1
PARKIN
MFN2
p-PARKIN
DRP1
PGC1α
P-DRP1
TOM20
GAPDH

Figure 3G

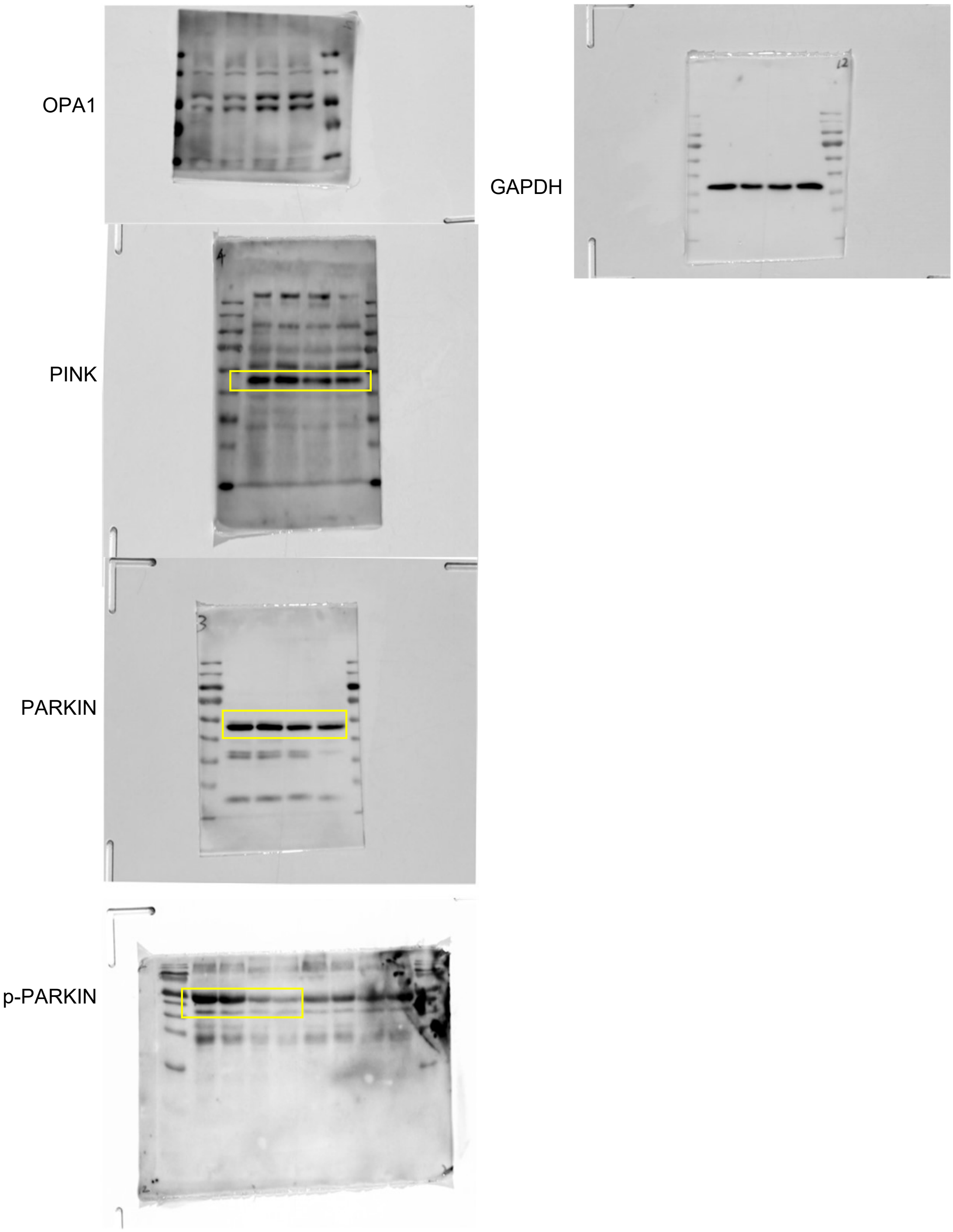

Figure 4 A

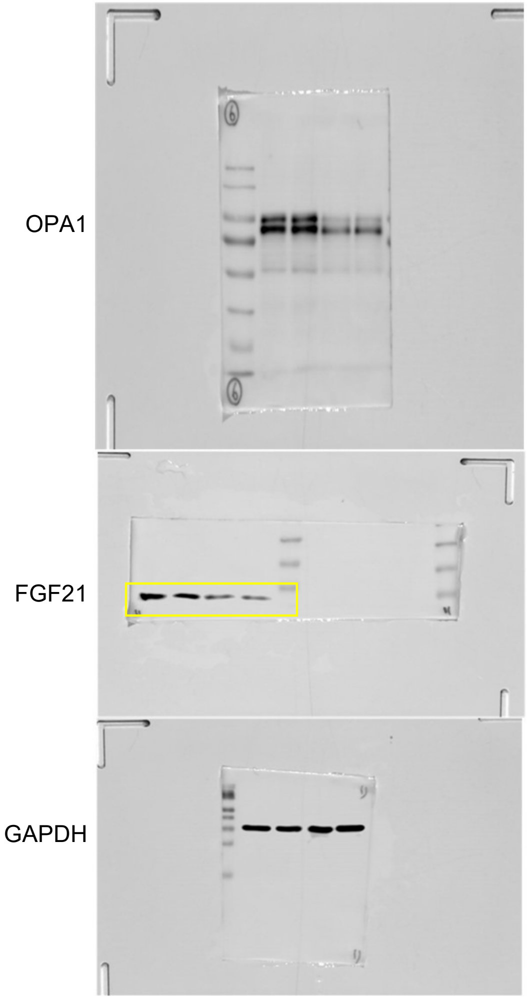

D

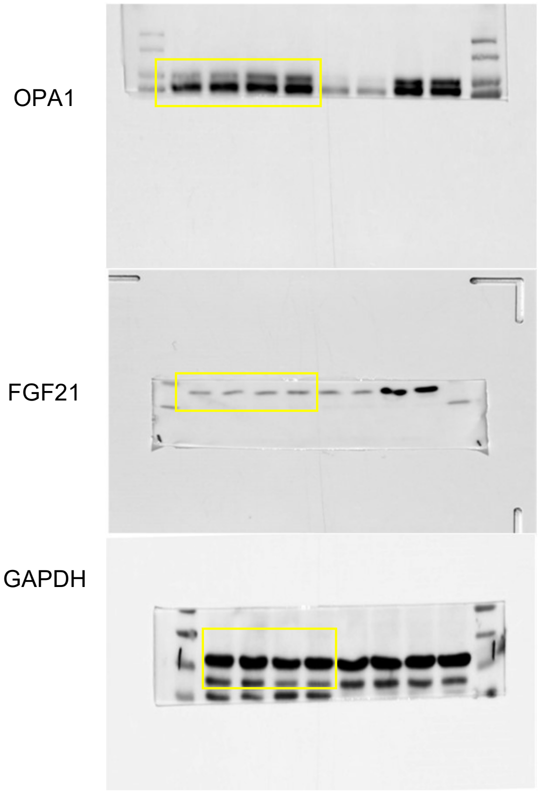

E

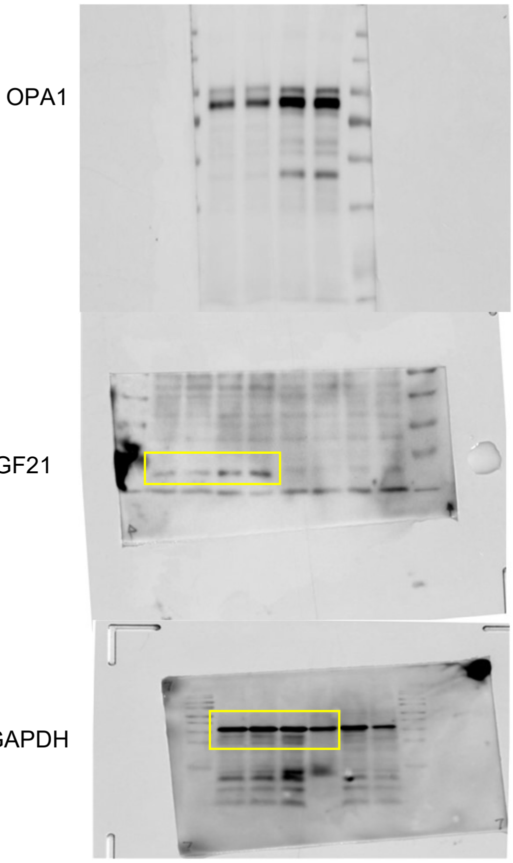

Figure 5A

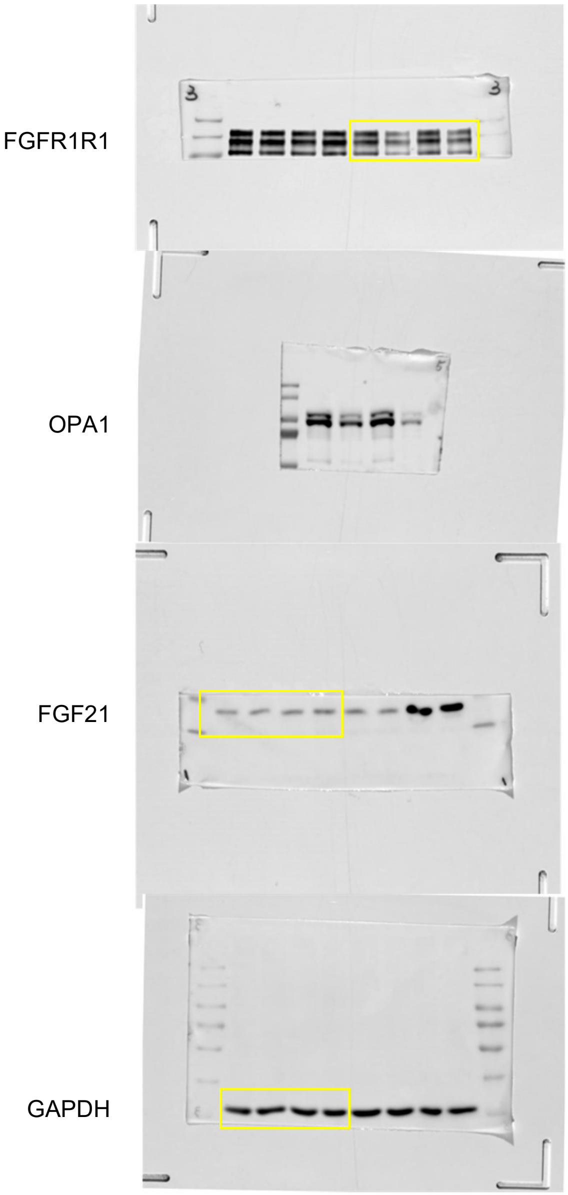

Figure 6H

OPA1

GAPDH

PINK

PARKIN

p-PARKIN

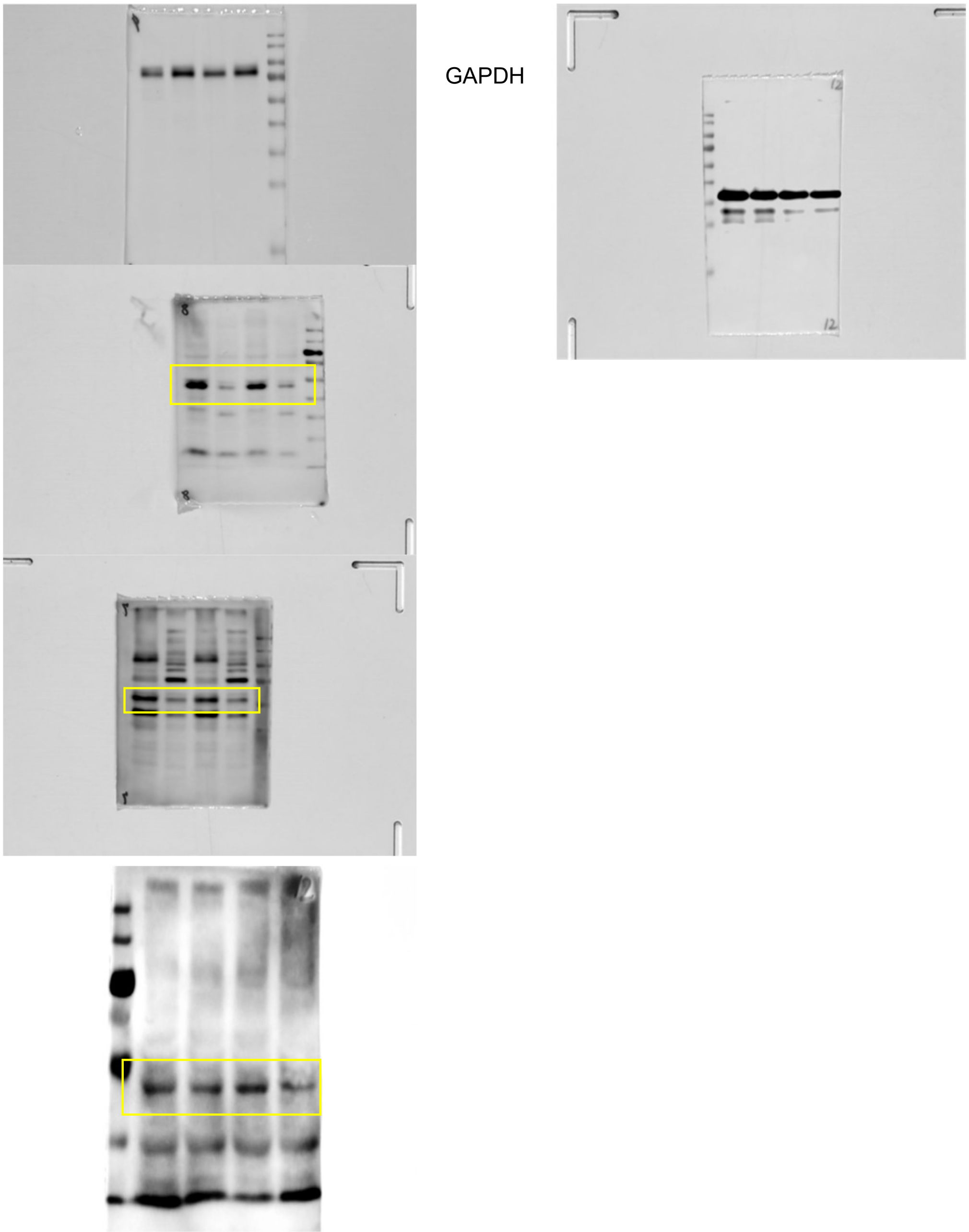

Figure 7A

OPA1

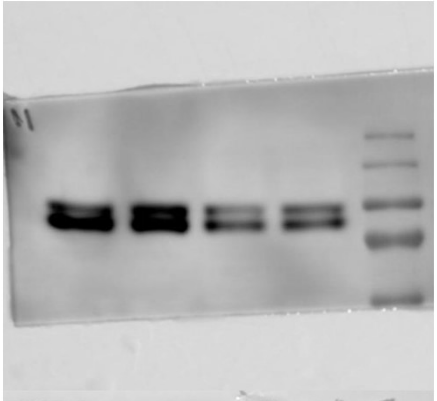

GAPDH

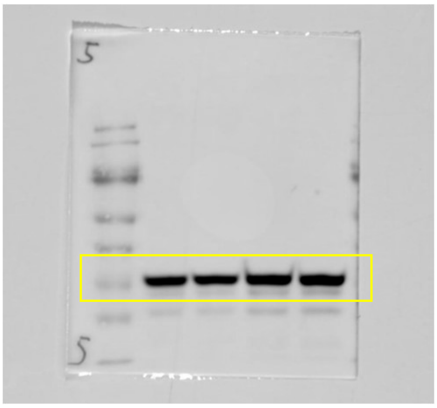

PINK

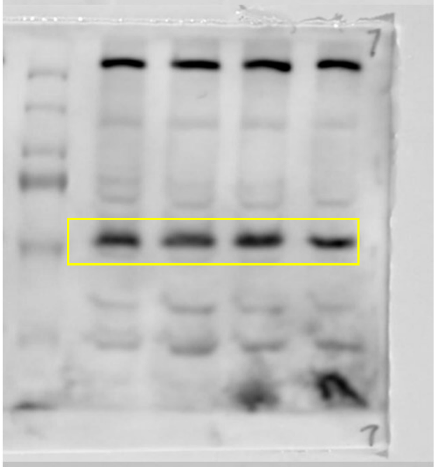

PARKIN

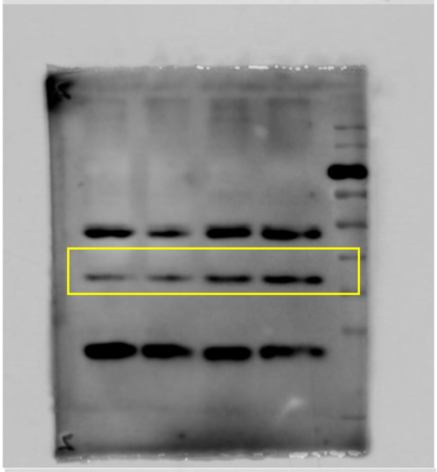

p-PARKIN

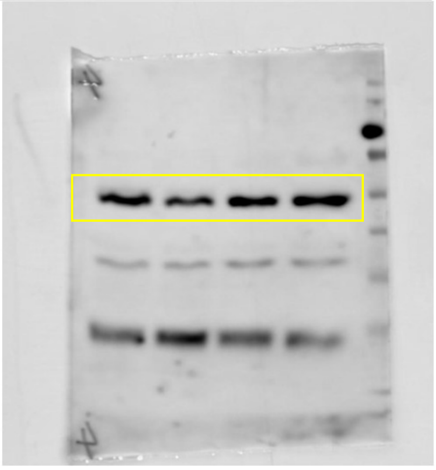

Figure 8

A

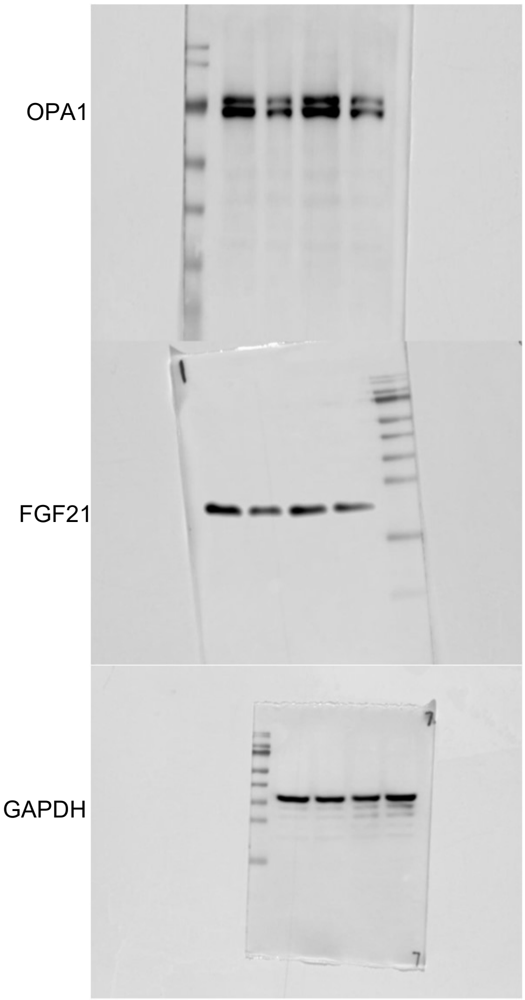

B

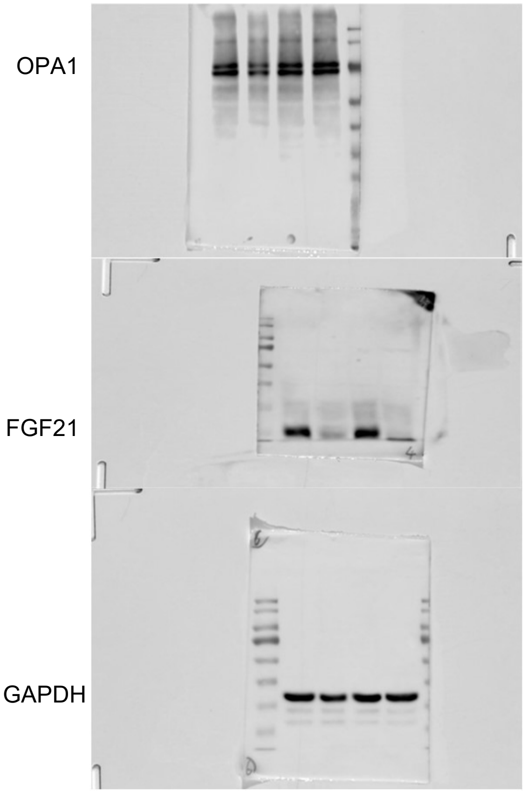

Figure 8

C

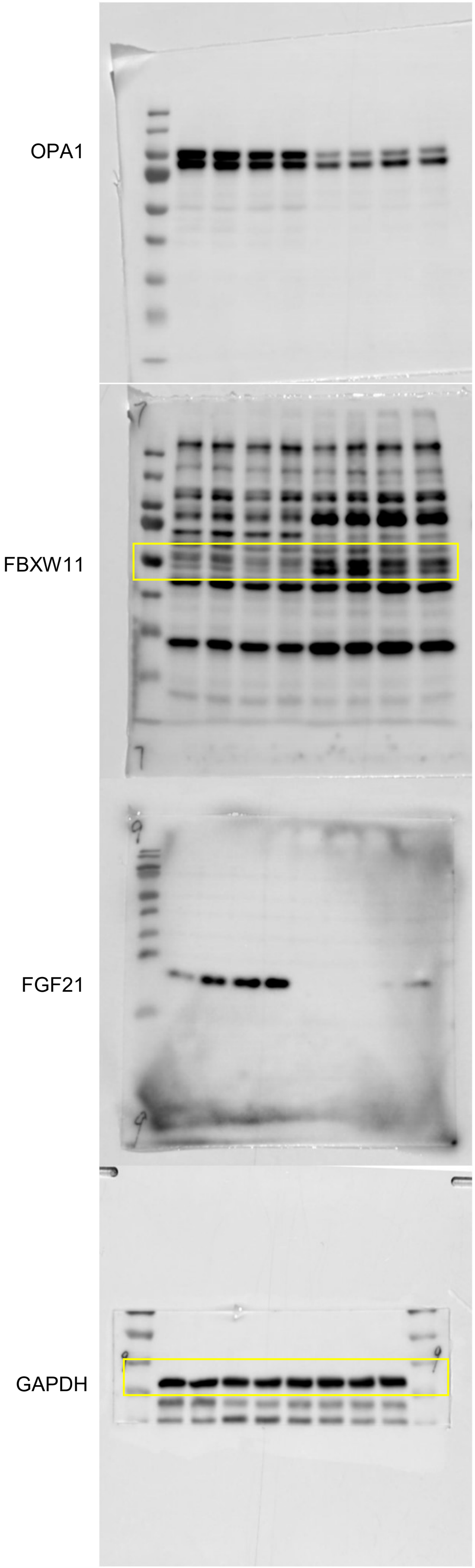

Figure 8

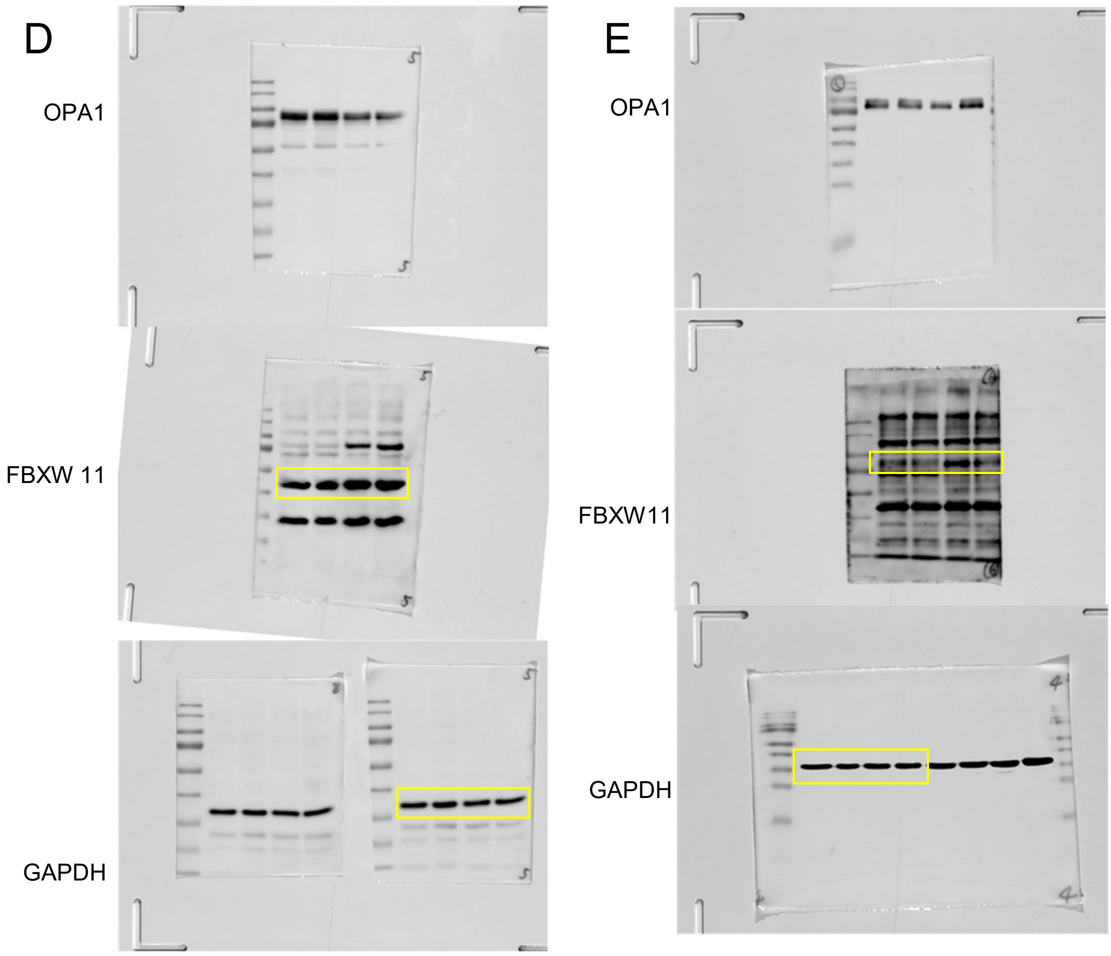

Figure 8F

IP

His

Flag

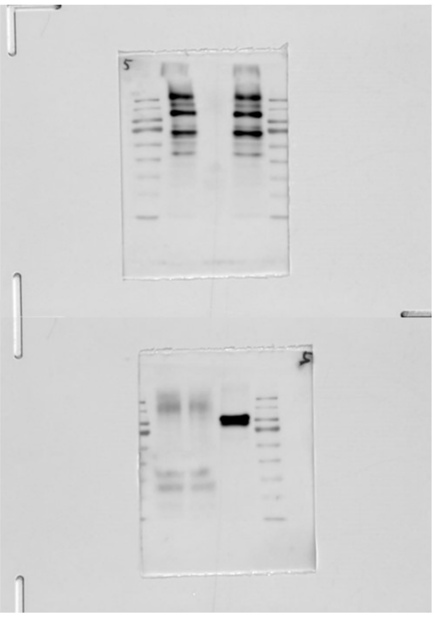

Input

His

Flag

GAPDH

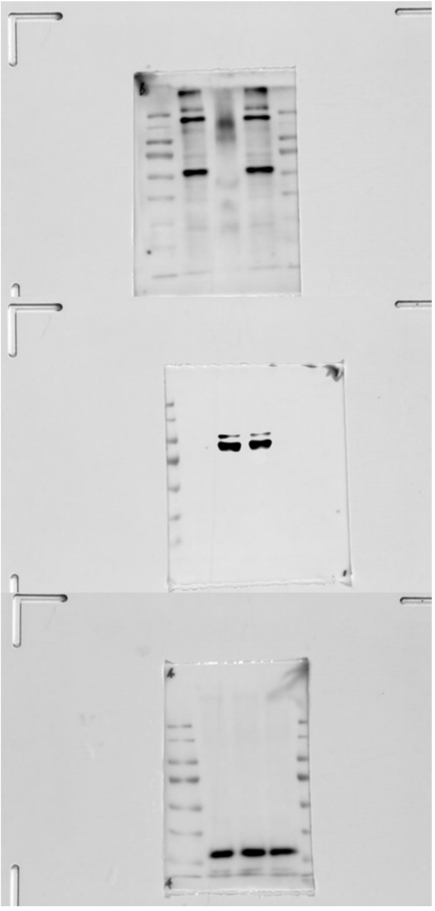

Figure 8G

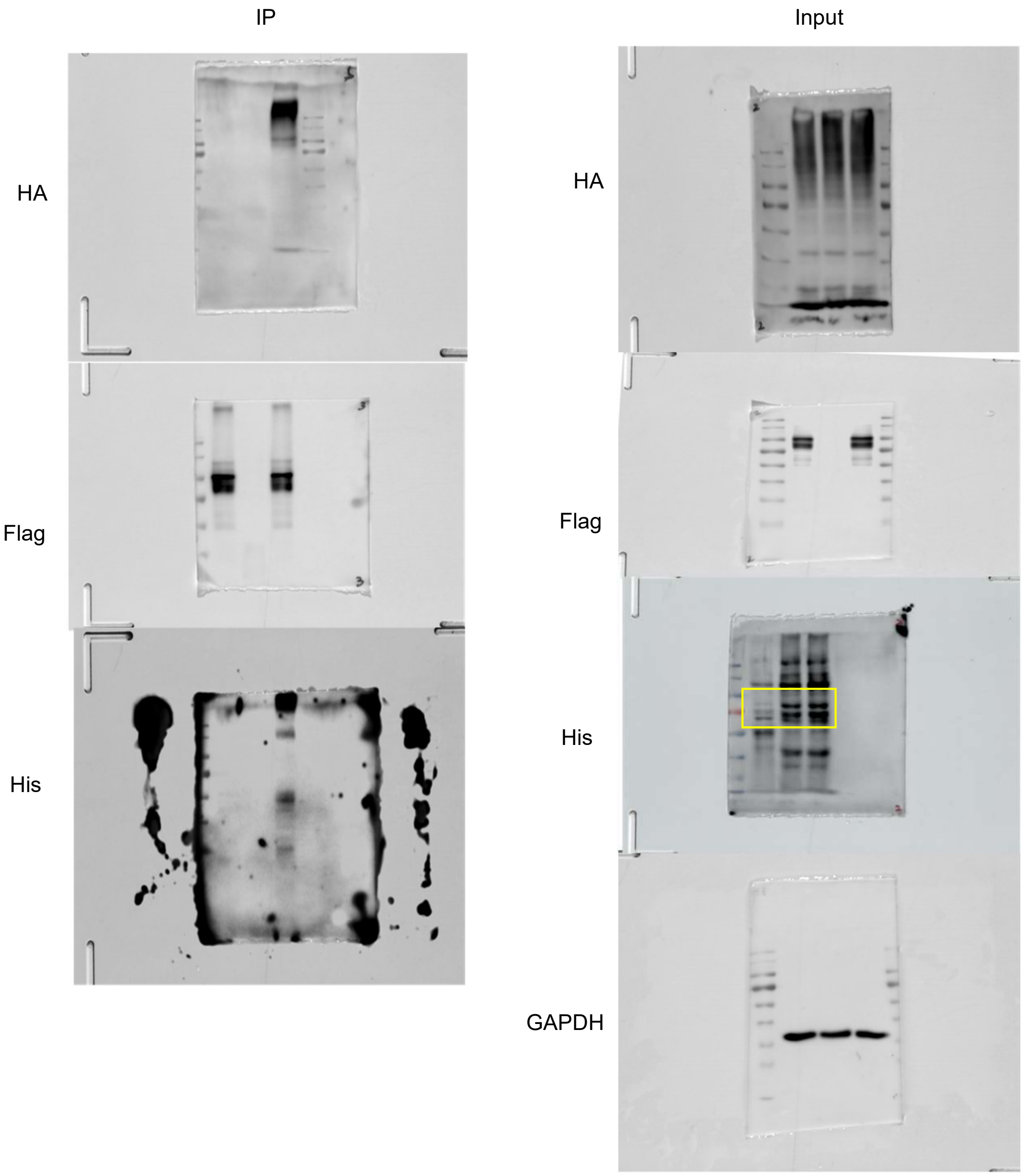

Supplement: Supplementary file 2 — Full and uncropped western blots [file 41419_2023_5842_MOESM2_ESM.pdf]
