# Supplementary material for: FGF21-FGFR1 controls mitochondrial homeostasis in cardiomyocytes by modulating the degradation of OPA1
Source: Cell Death Dis. 2023 May 8;14(5):311. doi: 10.1038/s41419-023-05842-9 (PMC10167257; doi:10.1038/s41419-023-05842-9)
Supplement: Supplementary file 3 — Reproducibility checklist [file 41419_2023_5842_MOESM3_ESM.pdf]

Corresponding Author Name: [illegible]
Manuscript Number: [illegible]

SPRINGER NATURE

Reporting Summary

Springer Nature wishes to improve the reproducibility of the work that we publish. This checklist is used to ensure good reporting standards and to improve the reproducibility. Please respond completely to all questions relevant to your manuscript. For more information, please read the journal's Guide to Authors.

☐ **Check here to confirm that the following information is available in the Material & Methods section:**

- **The exact sample size (*n*)** for each experimental group/condition, given as a number, not a range
- **A description of the sample collection** allowing the reader to understand whether the samples represent technical or biological replicates (including how many animals, litters, culture, etc.)
- **A statement of how many times the experiment shown was replicated in the laboratory**
- **Definitions of statistical methods and measures**: For small sample sizes (n<5) descriptive statistics are not appropriate, instead plot individual data points
    - Very common tests, such as *t*-test, simple χ^2 tests, Wilcoxon and Mann-Whitney tests, can be unambiguously identified by name only, but more complex techniques should be described in the methods section
    - Are tests one-sided or two-sided?
    - Are there adjustments for multiple comparisons?
    - **Statistical test results**, e.g., ***P* values**
    - Definition of **'center values'** as **median or mean**;
    - Definition of **error bars as s.d. or s.e.m. or c.i.**

Please ensure that the answers to the following questions are reported in the manuscript itself. We encourage you to include a specific subsection in the methods section for statistics, reagents and animal models. Below, provide the page number or section and paragraph number.

Statistics and general methods

	Reported in section/paragraph or page #
1. How was the sample size chosen to ensure adequate power to detect a pre-specified effect size? (Give section/paragraph or page #)	Section:MATERIALS AND METHODS, Statistical analysis/ Page #18.
For animal studies, include a statement about sample size estimate even if no statistical methods were used.	Section:MATERIALS AND METHODS, Statistical analysis/ Page #18.
2. Describe inclusion/exclusion criteria if samples or animals were excluded from the analysis. Were the criteria pre-established? (Give section/paragraph or page #)	Section:MATERIALS AND METHODS, Statistical analysis/ Page #18.
3. If a method of randomization was used to determine how samples/animals were allocated to experimental groups and processed, describe it. (Give section/paragraph or page #)	Section:MATERIALS AND METHODS, Statistical analysis/ Page #18.
For animal studies, include a statement about randomization even if no randomization was used.	Section:MATERIALS AND METHODS, Statistical analysis/ Page #18.

4. If the investigator was blinded to the group allocation during the experiment and/or when assessing the outcome, state the extent of blinding. (Give section/paragraph or page #)	Section: MATERIALS AND METHODS / Page #18.
For animal studies, include a statement about blinding even if no blinding was done.	Section: MATERIALS AND METHODS / Page #18.
5. For every figure, are statistical tests justified as appropriate?	Section: FIGURE LEGENDS
Do the data meet the assumptions of the tests (e.g., normal distribution)?	Section: FIGURE LEGENDS
Is there an estimate of variation within each group of data?	Section: FIGURE LEGENDS
Is the variance similar between the groups that are being statistically compared? (Give section/paragraph or page #)	Section: FIGURE LEGENDS

Reagents

Reported in section/paragraph or page #

6. Report the source of antibodies (vendor and catalog number)	Section: MATERIALS AND METHODS/ Page #24.
7. Identify the source of cell lines and report if they were recently authenticated (e.g., by STR profiling) and tested for mycoplasma contamination	Section: MATERIALS AND METHODS/ Page #24.

Animal Models

Reported in section/paragraph or page #

8. Report species, strain, sex and age of animals	Section: MATERIALS AND METHODS/ Page #18.
9. For experiments involving live vertebrates, include a statement of compliance with ethical regulations and identify the committee(s) approving the experiments.	Section: MATERIALS AND METHODS/ Page #18.

10. We recommend consulting the ARRIVE guidelines (*PLoS Biol.* **8**(6), e1000412,2010) to ensure that other relevant aspects of animal studies are adequately reported.

Human subjects

Item	Reported in section/paragraph or page #
11. Identify the committee(s) approving the study protocol.	N/A
12. Include a statement confirming that informed consent was obtained from all subjects.	N/A
13. For publication of patient photos, include a statement confirming that consent to publish was obtained.	N/A
14. Report the clinical trial registration number (at ClinicalTrials.gov or equivalent).	N/A

15. For phase II and III randomized controlled trials, please refer to the CONSORT statement and submit the CONSORT checklist with your submission.

16. For tumor marker prognostic studies, we recommend that you follow the REMARK reporting guidelines.

Data deposition

Item	Reported in section/paragraph or page #
17. Provide accession codes for deposited data. Data deposition in a public repository is mandatory for:<br>a. Protein, DNA and RNA sequences<br>b. Macromolecular structures<br>c. Crystallographic data for small molecules<br>d. Microarray data	N/A

Deposition is strongly recommended for many other datasets for which structured public repositories exist; more details on our data policy are available in the Guide to Authors. We encourage the provision of other source data in supplementary information or in unstructured repositories such as Figshare and Dryad. We encourage publication of Data Descriptors (see Scientific Data) to maximize data reuse.

Item	Reported in section/paragraph or page #
18. If computer code was used to generate results that are central to the paper's conclusions, include a statement in the Methods section under "**Code availability**" to indicate whether and how the code can be accessed. Include version information as necessary and any restrictions on availability.	N/A
